# Mutations in mitochondrial ferredoxin FDX2 suppress frataxin deficiency

**DOI:** 10.1038/s41586-025-09821-2

**Published:** 2025-12-10

**Authors:** Joshua D. Meisel, Pallavi R. Joshi, Amy N. Spelbring, Hong Wang, Sandra M. Wellner, Presli P. Wiesenthal, Maria Miranda, Jason G. McCoy, David P. Barondeau, Gary Ruvkun, Vamsi K. Mootha

**Affiliations:** 1 Department of Molecular Biology, Massachusetts General Hospital, Boston, MA USA; 2 Harvard Medical School, Boston, MA USA; 3 Broad Institute, Cambridge, MA USA; 4 Howard Hughes Medical Institute, Massachusetts General Hospital, Boston, MA USA; 5 Department of Biology, Brandeis University, Waltham, MA USA; 6 Department of Chemistry, Texas A&M University, College Station, TX USA

**Keywords:** Metals, Mechanisms of disease, Mitochondria, Epistasis

## Abstract

Frataxin is a key component of an ancient, mitochondrial iron–sulfur cluster biosynthetic machinery, serving as an allosteric activator of the cysteine desulfurase NFS1 (refs. ^1–5^). Loss of frataxin levels underlies Friedreich’s ataxia^6^, the most common inherited ataxia. Yeast, *Caenorhabditis elegans* and human cells can tolerate loss of frataxin when grown in ‘permissive’ low oxygen tensions^7^. Here we conducted an unbiased, genome-scale forward genetic screen in *C. elegans* leveraging permissive and non-permissive oxygen tensions to discover suppressor mutations that bypass the need for frataxin. All mutations act dominantly and are in the ferredoxin *FDX2*/*fdx-2* or in the cysteine desulfurase *NFS1*/*nfs-1* genes, resulting in amino-acid substitutions at the FDX2–NFS1 binding interface. Our genetic and biochemical analyses show that the suppressor mutations boost iron–sulfur cluster levels in the absence of frataxin. We also demonstrate that an excess of FDX2 inhibits frataxin-stimulated NFS1 activity in vitro and blocks the synthesis of iron–sulfur clusters in mammalian cell culture. These findings are consistent with structural and biochemical evidence that frataxin and FDX2 compete for occupancy at the same site on NFS1 (refs. ^8,9^). We show that lowering levels of wild-type FDX2 through loss of one gene copy can ameliorate the growth of frataxin mutant *C. elegans* or the ataxia phenotype of a mouse model of Friedreich’s ataxia under normoxic conditions. These genetic and biochemical studies indicate that restoring the stoichiometric balance of frataxin and FDX2 through partial knockdown of *FDX2* may be a potential therapy for Friedreich’s ataxia.

## Abstract (web summary)

A forward genetic screen in *Caenorhabditis elegans* reveals that decreased levels of the ferredoxin FDX2 suppress the loss of frataxin in worms and in mice by relief of FDX2 inhibition of frataxin-stimulated NFS1 activity.

## Main

Iron–sulfur (Fe–S) clusters are ancient and essential cofactors necessary for the activity of dozens of proteins in the cell, including those involved in the electron transport chain (ETC), tricarboxylic acid cycle, DNA repair in the nucleus and protein translation in the cytosol^10^. Synthesis of Fe–S clusters occurs in the mitochondrial matrix by an enzyme-mediated pathway consisting of the scaffold protein ISCU2, the cysteine desulfurase NFS1 that provides sulfur from cysteine, LYRM4 and NDUFAB1 (the acyl carrier protein) that stabilize the complex, the ferredoxin FDX2 that conveys electrons from NADPH (the reduced form of nicotinamide adenine dinucleotide phosphate) and ferredoxin reductase (FDXR), and frataxin^11,12^. Frataxin is an allosteric activator of NFS1 (ref. ^2^), proposed to accelerate the formation of [2Fe–2S] clusters by promoting persulfide transfer from NFS1 to ISCU2 (refs. ^1,3–5^).

Decreased levels or activity of frataxin underlies Friedreich’s ataxia^6^, which affects 1 in 50,000 people, making it the most common monogenic mitochondrial disease and the most common recessive ataxia. Friedreich’s ataxia presents with ataxia, cardiomyopathy and increased incidence of diabetes; patients have an average lifespan of 37.5 years. Knockout of frataxin causes lethality in mice and *Caenorhabditis elegans*^13,14^, but we have shown that growth of yeast, *C. elegans* and human cells lacking frataxin can be rescued by hypoxia due to an increase in Fe–S cluster levels^7^. Our ability to culture frataxin-null mutant *C. elegans* in ‘permissive’ hypoxic environments affords us the unique opportunity to screen for rare bypass mutations that have been identified in yeast^15^ but never in animals. Our approach is analogous to the classical use of temperature-sensitive mutants that can be grown in low-temperature environments but then experience fitness defects at non-permissive higher temperatures^16^. Genetic suppressors of frataxin deficiency could shed light on the specific role of frataxin in Fe–S cluster biogenesis and the mechanism of hypoxia rescue while revealing new therapeutic targets.

Here, we use *C. elegans* forward genetics to discover dominant-acting missense mutations in *FDX2/fdx-2* and *NFS1/nfs-1* that rescue the growth and development defects of frataxin-null animals, in a manner additive with hypoxia. Our suppressor mutations partially restore Fe–S cluster production, leading to increased levels of ETC complexes, which contributes to the growth rescue. Using in vitro biochemistry and mammalian cell culture, we show that excess FDX2 binding to the iron–sulfur assembly complex is inhibitory to Fe–S cluster production. Reduced occupancy of FDX-2 on NFS-1, achieved either through missense mutations at the binding interface or decreased levels of wild-type FDX-2 in a heterozygous *fdx-2* null mutant, partially bypasses the need for frataxin. Finally, we demonstrate that reducing *Fdx2* expression can alleviate neurological defects observed in a mouse model of Friedreich’s ataxia.

## Results

**Fig. 1:**
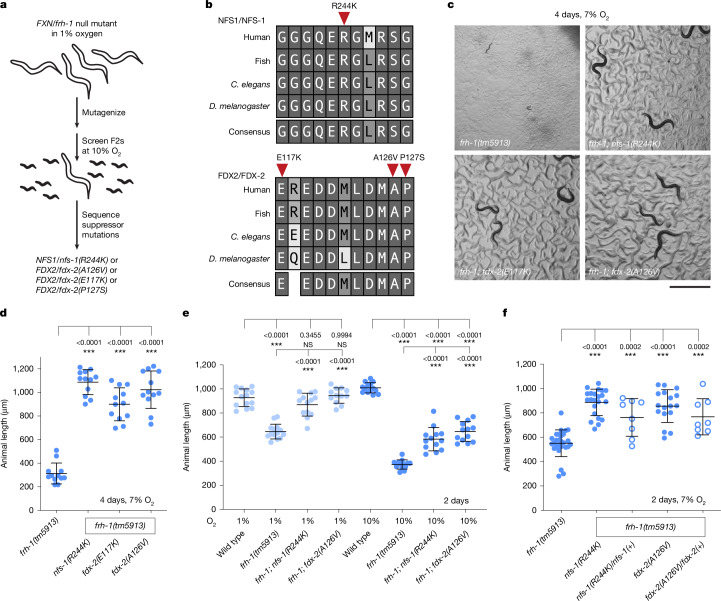
Mutations in *NFS1/nfs-1* or *FDX2/fdx-2* partially rescue the growth defect caused by frataxin loss in *C. elegans.* **a**, A forward genetic screen using random chemical mutagenesis revealed particular substitution mutations in *fdx-2* and *nfs-1* that can rescue the loss of frataxin. **b**, Multiple sequence alignment of NFS1 (*C. elegans* residues 239–251) and FDX2 (*C. elegans* residues 117–127) including homologues from mammals, fish and invertebrates made using ClustalW. **c**, Synchronized frataxin-null animals grown at 7% oxygen for 4 days with or without suppressor mutations in *fdx-2* and *nfs-1*. Scale bar, 1 mm. **d**–**f**, Growth of *C. elegans* at 7% oxygen (4 days) (**d**), 1% or 10% oxygen (2 days) (**e**) or 7% oxygen (2 days) (**f**) quantified by body length measurements. The number of individual worms in each group was: all groups *n* = 12 (**d**), all groups *n* = 12–14 (**e**), *frh-1* *n* = 27, *frh-1; nfs-1* *n* = 22, *frh-1; nfs-1/+* *n* = 8, *frh-1; fdx-2* *n* = 17, *frh-1; fdx-2/+* *n* = 8 (**f**). For all panels, statistical significance was calculated using one-way analysis of variance (ANOVA) followed by Dunnett’s (**d**,**f**) or Sidak’s (**e**) multiple comparison test. Error bars represent mean ± s.d. NS, not significant, *P < 0.05, **P < 0.01, ***P < 0.001.

Using the ability to cultivate frataxin/*frh-1* null mutant *C. elegans* in hypoxia, we performed a forward genetic selection for rare suppressor mutations that allow *frh-1* null mutant animals to grow at what is normally a non-permissive oxygen tension (Fig. 1a). *frh-1(tm5913)* animals carrying a deletion in the frataxin gene were grown at 1% oxygen, a permissive condition under which they are viable, and randomly mutagenized, generating hundreds of new mutations throughout the genome in each of thousands of animals. Two generations later, tens of thousands of F_2_ progeny were transferred to 10% oxygen and challenged to grow at this non-permissive oxygen tension for a *frh-1* null mutant. Whereas most animals arrested development as larvae, such as the parental *frh-1(tm5913)* strain, rare animals carrying a newly induced suppressor mutation reached adulthood and were isolated. These mutants still carried a deletion in the frataxin gene. Following whole-genome sequencing, we identified that suppressor mutant strains also carried missense mutations in one of two genes: the cysteine desulfurase *nfs-1* (one allele corresponding to R244K) or the mitochondrial ferredoxin *Y73F8A.27* (three independent alleles corresponding to E117K, A126V and P127S) (Fig. 1b and Extended Data Table 1).

**Extended Data Fig. 1:**
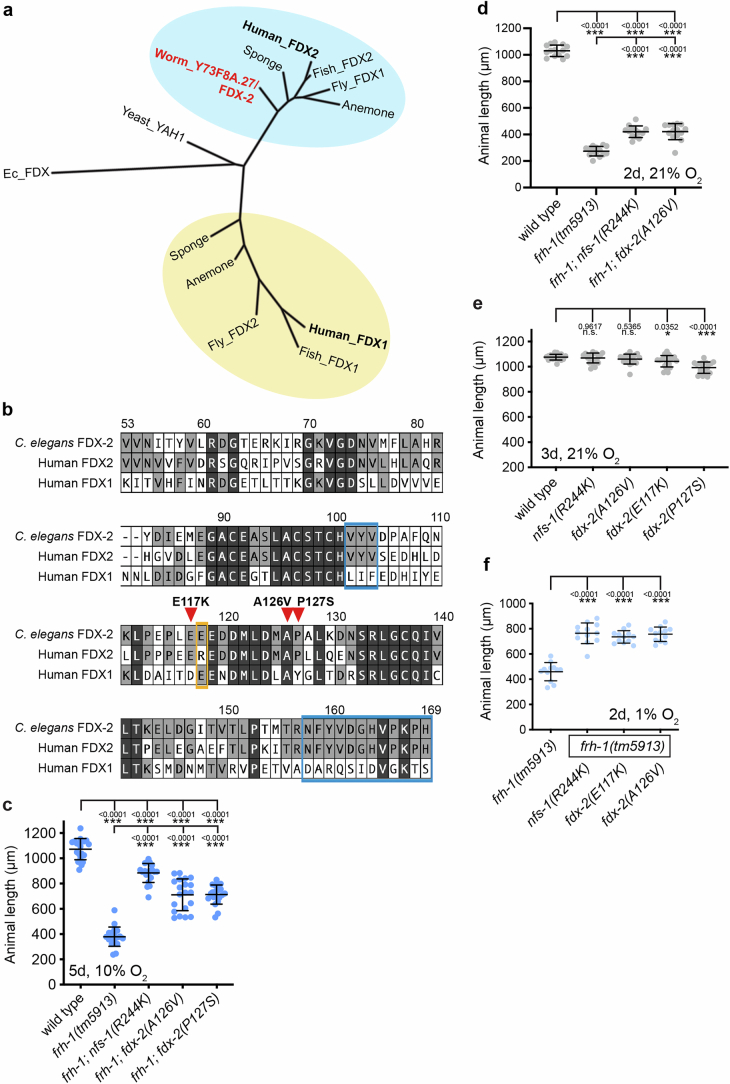
Mutations in *NFS1/nfs-1* or *FDX2/fdx-2* partially rescue the growth defect caused by frataxin loss in *C. elegans.* **a**, Phylogenetic tree of FDX1 and FDX2 homologs from *E. coli*, *S. cerevisiae*, *Amphimedon queenslandica*, *Nematostella vectensis*, *C. elegans, D. melanogaster, D. rerio*, and *H. sapiens*. Alignments were made with Clustal Omega, maximum-likelihood trees made using PhyML and visualized with FigTree. **b**, Multiple sequence alignment of *C. elegans* FDX-2 with human FDX2 and FDX1. Sequences necessary and sufficient for the activity of FDX2 are boxed in blue, and a residue critical for the activity of FDX1 is boxed in orange^19^. **c**,**d**,**e**,**f**, Growth of *C. elegans* at 10% oxygen (5 days) (**c**), 21% oxygen (2 days) (**d**), 21% oxygen (3 days) (**e**), or 1% oxygen (2 days) (**f**) quantified by body length measurements. The number of individual worms in each group was: all groups n = 20 (**c**), all groups n = 13-14 (**d**), all groups n = 20 (**e**), all groups n = 12 (**f**). For all panels statistical significance was calculated using one-way ANOVA followed by Dunnett’s (**e,f**) or Sidak’s (**c,d**) Multiple Comparison Test. Error bars represent mean ± standard deviation. n.s. = not significant, * = p value < 0.05, ** = p value < 0.01, *** = p value < 0.001.

Most metazoa encode two ferredoxin paralogues, FDX1 and FDX2, both of which harbour [2Fe–2S] clusters and are localized to mitochondria. FDX2 is required for de novo [2Fe–2S] cluster biosynthesis, donating electrons to a persulfide on the scaffold protein ISCU2 (refs. ^4,17,18^). FDX1 has long been known to be required for steroidogenesis and, more recently, has been shown to be key for the synthesis of haem *a* (for cytochrome *c* oxidase) and lipoyl synthase^19–22^. These ferredoxins cannot functionally complement each other due to sequence divergence that lends specificity to target substrates^18–21^. Although basal metazoa such as sponges and anemones encode both paralogues, *C. elegans* contain only one mitochondrial ferredoxin, *Y73F8A.27*, and through phylogenetic analysis we inferred that *C. elegans* retain FDX2 and have lost FDX1 (Extended Data Fig. 1a). Thus, *Y73F8A.27* has been renamed *fdx-2*. The alignment of *C. elegans* FDX-2 with human FDX2 and FDX1 revealed that *C. elegans* FDX-2 contains key sequence features^19^ of both FDX2 and FDX1 (Extended Data Fig. 1b), raising the possibility that it has acquired new FDX1-like functionality. Notably, all identified *nfs-1* and *fdx-2* suppressor mutations occur in highly conserved residues also present in human NFS1 and FDX2, respectively (Fig. 1b and Extended Data Table 1).

We used CRISPR–Cas9 to generate *nfs-1* and *fdx-2* mutant alleles in a clean genetic background and confirmed that *nfs-1(R244K)*,* fdx-2(E117K)*,* fdx-2(A126V)* or *fdx-2(P127S)* mutations improved the growth of *frh-1* null animals at non-permissive 7–10% oxygen (Fig. 1c–e and Extended Data Fig. 1c), which is the natural environmental oxygen tension for *C. elegans* nematodes^23^. Analysing frataxin-null animals carrying heterozygous mutations in *nfs-1(R244K)* or *fdx-2(A126V)* revealed that these suppressor mutations dominantly rescue growth of frataxin mutants (Fig. 1f). The point mutations in *nfs-1* and *fdx-2* also significantly improved the growth of *frh-1* mutants at the more severe 21% oxygen (Extended Data Fig. 1d). In a *frh-1(wt)* background the *nfs-1(R244K)* and *fdx-2(A126V)* point mutations caused no growth defect at 21% oxygen (Extended Data Fig. 1e), whereas the *fdx-2(E117K)* and *fdx-2(P127S)* point mutations produced small but significant effects on growth rate. At 1% oxygen, a permissive condition that supports the growth and fertility of frataxin mutants^7^, the *nfs-1* and *fdx-2* suppressor mutations further rescued the *frh-1* growth rate to wild-type levels (Fig. 1e and Extended Data Fig. 1f). Taken together, these results identify suppressor mutations within the Fe–S cluster assembly complex that partially rescue the growth and developmental defects of frataxin loss.

**Fig. 2:**
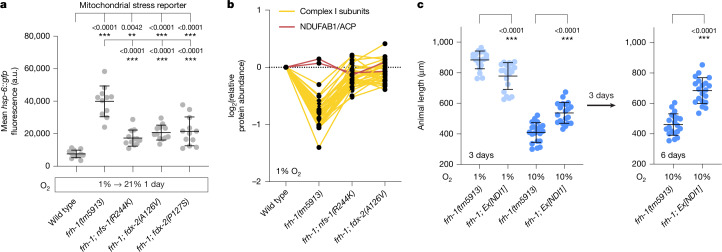
Frataxin suppressor mutations restore levels of Fe–S cluster-containing ETC complexes. **a**, Mean intestinal fluorescence of age-matched day 1 adult animals containing *hsp-6::gfp* exposed to 21% oxygen for 24 h. The number of individual worms in each group was *n *= 11–12. **b**, Quantitative TMT proteomics of complex I subunits in wild-type animals, frataxin mutants and frataxin mutants with suppressor mutations *nfs-1(R244K) or fdx-2(A126V)* grown continuously at 1% oxygen. Values are normalized to wild type; each line represents one protein from *n* = 1 experiment. Proteins shown from which at least two peptides were quantified. **c**, Growth of *C. elegans* at 1% or 10% oxygen for indicated durations. The number of individual worms in each group was *n* = 20. For all panels, statistical significance was calculated using one-way ANOVA followed by Sidak’s multiple comparison test (**a**,**c**). Error bars represent mean ± s.d. *P < 0.05, **P < 0.01, ***P < 0.001. a.u., arbitrary units.

**Extended Data Fig. 2:**
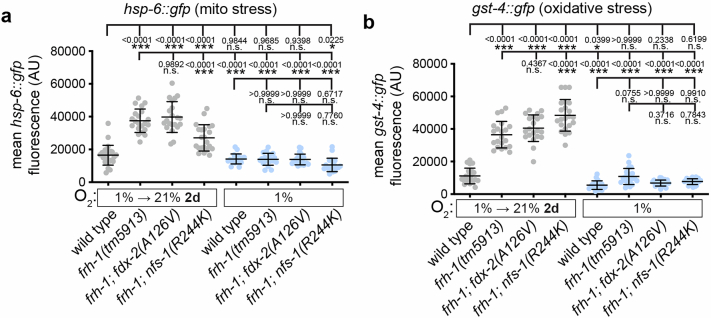
Frataxin suppressor mutations do not fully alleviate stress-response pathway induction. **a,b**, Mean intestinal fluorescence of age-matched day 2 adult animals containing *hsp-6::gfp* (**a**) or *gst-4::gfp* (**b**) grown continuously at 1% oxygen or exposed to 21% oxygen for 48 h. The number of individual worms in each group was: all groups n = 19-26 (**a**), all groups n = 21-24 (**b**). For all panels statistical significance was calculated using one-way ANOVA followed by Sidak’s Multiple Comparison Test (**a,b**). Error bars represent mean ± standard deviation. n.s. = not significant, * = p value < 0.05, ** = p value < 0.01, *** = p value < 0.001.

To understand how the *nfs-1* and *fdx-2* suppressor mutations rescue the growth defects of frataxin-null animals, we first analysed transcriptional reporters for *C. elegans* stress responses. Both the *hsp-6::gfp* reporter for mitochondrial stress and the *gst-4::gfp* reporter for oxidative stress were elevated in the *frh-1* mutant at 21% oxygen but not induced at 1% oxygen^7^ (and Extended Data Fig. 2a,b). The striking rescue by hypoxia may result from increased Fe–S cluster production and stability^7,24^, as well as the ability of hypoxia to rescue downstream pathways dependent on Fe–S clusters such as the ETC^25–27^ and lipoylation^20^. Similarly, induction of *hsp-6::gfp* fluorescence, which is a sensitive readout of mitochondrial membrane potential and protein import efficiency^28^, was partially decreased by *fdx-2* and *nfs-1* mutation in the *frh-1* null background (Fig. 2a and Extended Data Fig. 2a), suggesting an improvement in mitochondrial integrity. By contrast, the *fdx-2* and *nfs-1* suppressor mutations did not change the induction of *gst-4::gfp* fluorescence (Extended Data Fig. 2b), arguing against a global hypoxia-like restoration of ISCs to wild-type levels.

**Extended Data Fig. 3:**
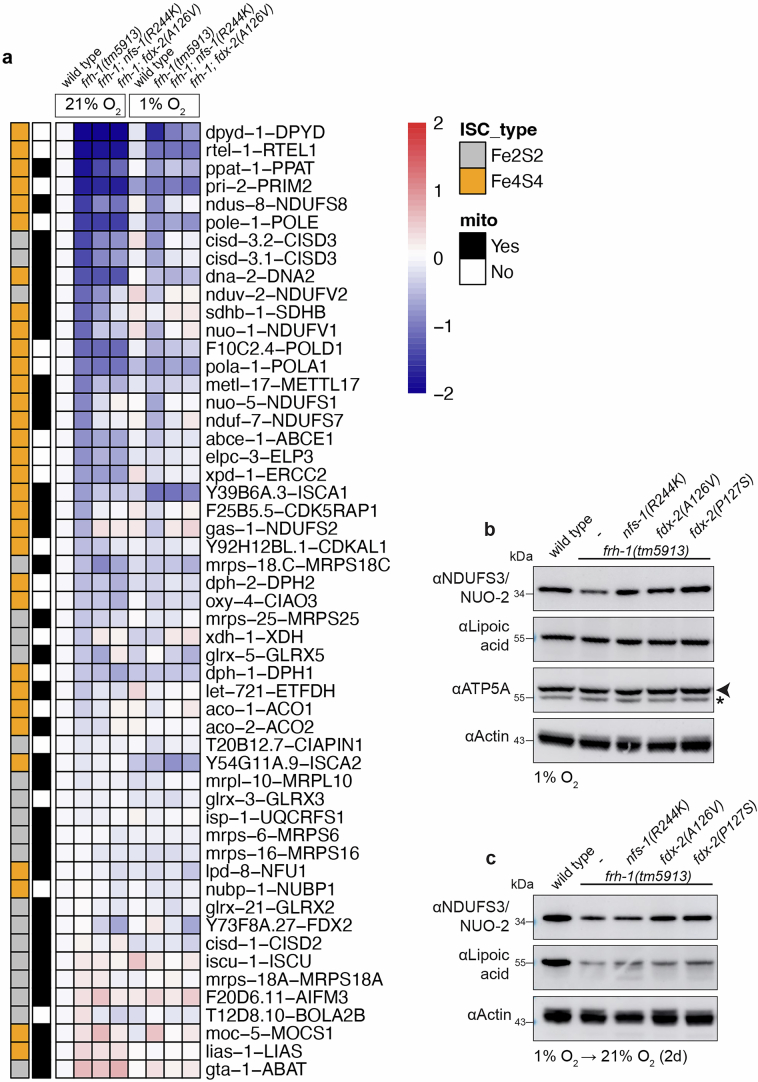
Frataxin suppressor mutations restore levels of Fe–S cluster-containing ETC complexes. **a**, TMT quantitative proteomics from animals grown continuously at 1% oxygen or animals shifted from 1% to 21% for 2 days. Plotted are log 2-fold ratios relative to wild type at 21% oxygen of all proteins from which at least two peptides were quantified. Fe–S cluster (also known as ISC)-containing proteins are annotated based on Andreini et al.^10^. supplemented with recently identified new Fe–S cluster proteins^29,54^. n = 1 experimental replicate. **b,c**, SDS-PAGE followed by western blot of whole worm lysate from animals exposed to continuous 1% oxygen (**b**) or shifted from 1% to 21% for 2 days (**c**). Arrow indicates ATP5A, star indicates non-specific band. For gel source data, see Supplementary Data 1.

To identify compromised cellular processes in the frataxin mutant that are rescued by *nfs-1* or *fdx-2* mutation, we performed quantitative tandem mass tag (TMT) proteomics in animals incubated continuously at 1% oxygen or animals shifted from 1% to 21% oxygen for 2 days. Analysis of Fe–S cluster-containing proteins revealed that *C. elegans* frataxin mutants shifted from 1% to 21% oxygen tended to be depleted for [4Fe–4S] proteins whereas [2Fe–2S] proteins were largely unaffected (Extended Data Fig. 3a and Supplementary Table 1). Following prolonged (10-day) loss of frataxin in cell culture virtually all Fe–S cluster proteins are depleted^29^. The *C. elegans* proteomics may reflect differences in half-life between [4Fe–4S]-containing and [2Fe–2S]-containing proteins after 2 days of Fe–S cluster biogenesis interruption. Of the 23 Fe–S cluster-containing proteins whose levels were depleted by at least 1.5-fold, hypoxia partially increased the levels of 21 out of 23 proteins, whereas the *fdx-2(A126V)* and *nfs-1(R244K)* suppressor mutations restored levels of only 10 Fe–S containing proteins, which included subunits of ETC complex I and complex II (Extended Data Fig. 3a).

In *C. elegans*, mutations in core subunits of complex I can destabilize the entire complex, resulting in the loss of other individual proteins^27^. Indeed, we found that all complex I subunits (except for the acyl carrier protein NDUFAB1) were depleted in *frh-1* mutants and rescued by *fdx-2* or *nfs-1* suppressor mutations (Fig. 2b). We confirmed these proteomics results with western blots against complex I subunit NDUFS3 (Extended Data Fig. 3b,c), which showed that at 1% or 21% oxygen frataxin loss caused low levels of complex I that were partially rescued by *fdx-2* or *nfs-1* point mutation. To determine whether ETC rescue was sufficient to improve the growth of frataxin mutants we expressed the yeast NADH dehydrogenase NDI1 (ref. ^30^), a single polypeptide that can bypass complex I and complex II in *C. elegans*^27,31,32^. By conveying electrons from NADH directly to ubiquinone, NDI1 is able to restore NADH redox balance as well as oxygen consumption and proton pumping by complexes III and IV. Expression of NDI1 was sufficient to partially rescue the growth of *frh-1* mutants (Fig. 2c). Taken together, these results indicate *fdx-2* and *nfs-1* point mutations partially restore Fe–S cluster biosynthesis in the absence of frataxin, and that the resulting increased flux through the ETC may be paramount to the rescue of animal growth and development.

**Fig. 3:**
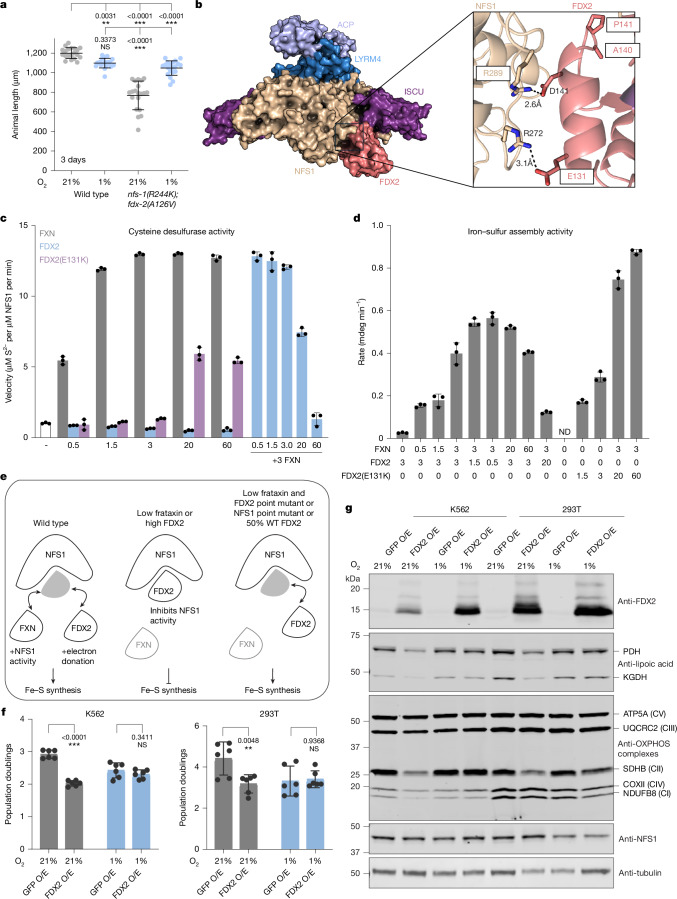
Excess FDX2 is detrimental to NFS1 activity and Fe–S cluster biosynthesis. **a**, Growth of animals for 3 days at room temperature exposed to 21% or 1% oxygen. The number of individual worms in each group was *n *= 20. **b**, Cryo-EM structure of the human Fe–S cluster assembly complex containing FDX2 (ref. ^9^) (Protein Data Bank 8RMC) with boxes indicating homologous residues to *C. elegans* suppressor mutations. **c**, Cysteine desulfurase activities of SDA_ec_ (0.5 µM; NFS1–ISD11–ACP_ec_) complexes containing ISCU2 (1.5 µM; white) and increasing equivalents of FXN (0.25–30 µM; grey), FDX2_ox_ (0.25–30 µM, oxidized ferredoxin 2; blue) and FDX2_ox_ E131K (0.25–30 µM; purple). Reactions were initiated with 2 mM l-cysteine, quenched after 3 min and the sulfide was converted to methylene blue to determine activities. For all groups *n* = 3 independent experiments. **d**, Iron–sulfur assembly activities on ISCU2 (100 µM) for complexes containing SDA_ec_ (1 µM), FXN (0–60 μM), FDX2 or FDX2 E131K (0–60 μM), FDXR (1 μM) and NADPH (500 μM). Reactions were initiated with 500 μM l-cysteine, and the change in ellipticity at 430 nm was fit to a linear equation to determine the rate of Fe–S assembly on ISCU2. The numbers on the *x* axis represent protein equivalents compared with SDA_ec_. ND, not detectable. For all groups *n* = 3 independent experiments. The initial and final circular dichroism spectra and averaged change in ellipticity at 430 nm for determining the reaction rate for each experiment are shown in Extended Data Fig. 5a–c. **e**, In wild-type conditions frataxin and FDX2 compete for binding to NFS1, promoting persulfide transfer and donating electrons, respectively, at distinct steps in the biosynthesis of Fe–S clusters. In the context of low or zero frataxin, or FDX2 overexpression, Fe–S synthesis is blocked due to frataxin displacement by FDX2 and/or FDX2 directly inhibiting NFS1 activity. Point mutations that weaken the NFS1–FDX2 interaction, or simply lowering FDX2 levels to generate NFS1 unbound by FDX2, can partially restore Fe–S synthesis and support growth in *C. elegans*. **f**, Three-day growth assay with K562 or 293T cells overexpressing (O/E) GFP or* FDX2* cDNA, grown in 21% or 1% oxygen tensions. Experiments were conducted in three biological replicates in technical duplicate for a total *n* = 6. **g**, Immunoblots for FDX2, lipoic acid, OXPHOS, NFS1 and loading control tubulin in K562 (left) and 293T (right) cell lysates collected from **f**. For gel source data, see Supplementary Data 1. For western blotting *n* = 3 biological replicates; all individual replicates for lipoic acid are shown and quantified in Supplementary Data 2. Statistical significance was calculated using one-way (**a**) or two-way (**f**) ANOVA followed by Sidak’s multiple comparison test. Error bars represent mean ± s.d. *P < 0.05, **P < 0.01, ***P < 0.001.

**Extended Data Fig. 4:**
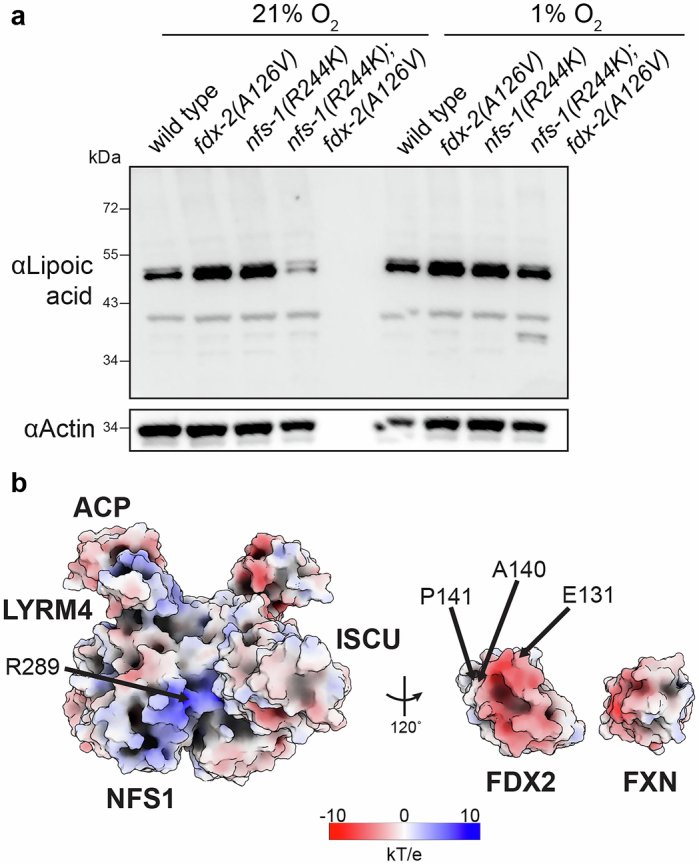
FDX2 and NFS1 suppressor mutation interact genetically and physically. **a**, SDS-PAGE followed by western blot of whole worm lysate from animals grown continuously at 1% oxygen or shifted from 1% to 21% for 2 days. For gel source data, see Supplementary Data 1. **b**, Cryo-EM structure of the human Fe–S cluster assembly complex containing FDX2^9^ (PDB: 8RMC) or FXN^33^ (PDB: 6NZU) with NFS1/FDX2 and NFS1/FXN interfaces exposed and electrostatic potential indicated.

To determine whether the *nfs-1* and *fdx-2* suppressor mutations were acting through a common mechanism we tested their genetic interaction. A double mutant of the *C. elegans* mutations *nfs-1(R244K); fdx-2(A126V)* in a frataxin wild-type background was synthetic sterile and grew slowly (Fig. 3a), despite each suppressor mutation on their own having no growth defect (Extended Data Fig. 1e). The *nfs-1(R244K); fdx-2(A126V)* double mutant also showed reduced lipoic acid staining (Extended Data Fig. 4a), consistent with reduced Fe–S cluster biosynthesis. The slow growth, sterility and low lipoic acid staining of the *nfs-1(R244K); fdx-2(A126V)* double mutant was rescued by hypoxia (Fig. 3a and Extended Data Fig. 4a), similar to other Fe–S cluster-deficient mutants and further supporting a loss of Fe–S cluster biosynthesis. Together, these results are consistent with the *nfs-1* and *fdx-2* suppressor mutations acting through the same pathway to rescue frataxin loss.

To understand the mechanism by which point mutations in *nfs-1* or *fdx-2* partially bypass the need for frataxin in Fe–S cluster biosynthesis, we mapped our newly identified suppressor mutations to recent cryogenic electron microscopy (cryo-EM) structures of the Fe–S cluster assembly complex. In structures containing frataxin^33^ we observed that NFS-1(R244) lies at the interface with frataxin, however our suppressor mutations were isolated in a frataxin-null background. Biochemical work in yeast has revealed that frataxin and the ferredoxin FDX2 interact with NFS1 at overlapping binding sites and can compete for binding to NFS1 (ref. ^8^), consistent with findings in bacteria^17,34^. Cryo-EM of the human Fe–S cluster assembly complex containing FDX2 confirmed that FDX2 binds NFS1 at the same interface as frataxin^9^. Indeed, the three suppressor mutations we isolated in FDX-2 (*C. elegans* E117K, A126V and P127S) lie on helix F at the NFS1–FDX2 interaction surface along with NFS-1(R244K) (Fig. 3b). Corresponding human residues NFS1(R289) and FDX2(E131) (Extended Data Table 1) form salt-bridges with FDX2(D141) and NFS1(R272), respectively, that stabilize the NFS1–FDX2 interaction^9^ (Fig. 3b and Extended Data Fig. 4b). Human residues FDX2(A140) and FDX2(P141) immediately follow FDX2 helix F and form the *i* and *i* + 1 residues of a type IV β turn. Proline frequently provides stabilization of β turns in the *i* + 1 position and the sidechain of alanine 140 faces into the core of the protein such that larger residues would sterically clash with the surrounding amino acids. Mutation of either of these residues would presumably lead to a change in the position of helix F and a disruption in the FDX2–NFS1 interface. We therefore propose that (1) all our suppressor mutations function similarly to perturb the NFS1–FDX2 interaction, and (2) in the absence of frataxin, ISC synthesis is partially inhibited due to constitutive binding of FDX2 to NFS1, and our suppressor mutations function to increase levels of the active form of the core NFS1–ISD11–ACP–ISCU2 Fe–S assembly complex.

To understand why FDX2 binding to NFS1—a necessary step in Fe–S cluster biosynthesis^17,18^—could be detrimental in the absence of frataxin (FXN), we measured the cysteine desulfurase activity of NFS1 in vitro using human proteins. Specifically, we measured the cysteine desulfurase activity of NFS1–ISD11–ACP_ec_ (SDA_ec_) complexes containing ISCU2 in the presence or absence of FXN and ox-FDX2. Increasing equivalents of FXN activated the core SDA_ec_U (SDA_ec_ plus ISCU2) Fe–S assembly complex (Fig. 3c, grey and Extended Data Table 2) in a similar manner as previously described^1^ with maximal velocity achieved with 3 equivalents. Unlike FXN, increased equivalents of wild-type FDX2 alone resulted in no stimulation in activity (Fig. 3c, blue). In the presence of FXN, when increasing equivalents of FDX2 were added (capable of displacing FXN^8^), NFS1 cysteine desulfurase activity was notably inhibited, resulting in kinetic parameters comparable to the SDA_ec_U complex (Fig. 3c, blue). Reactions with 0.5–3 equivalents of ox-FDX2 maintain high cysteine desulfurase activity suggesting that only excess FDX2 inhibits the FXN-based stimulation in sulfur transfer chemistry.

**Extended Data Fig. 5:**
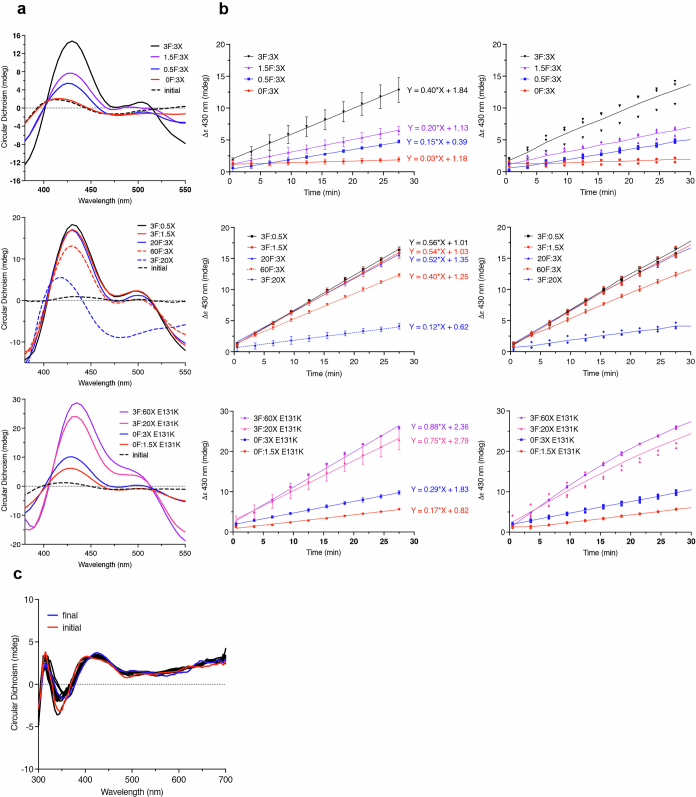
Excess FDX2 is detrimental to NFS1 activity and Fe–S cluster biosynthesis. **a**, Initial (dashed line) and final CD spectrum of Fe-S assembly reactions. **b**, Iron-sulfur assembly reactions on ISCU2 were monitored by the change in ellipticity at 430 nm. Spectra (300–700 nm) were measured every 3 min for 30 min, and the average change in ellipticity was plotted. The change in ellipticity at 430 nm was fit to a linear equation to determine the rate of Fe-S assembly on ISCU2. n = 3 independent experimental replicates. Numbers represent the equivalents of frataxin (F), and ferredoxin (X) compared to SDAec. Graphs display mean ± standard deviation (left) or individual data points (right). **c**, Raw CD spectrum of ISCU2 assembly reactions without X and F (initial spectrum, red; final spectrum, blue). Spectra were recorded every 3 min for 30 min (black lines).

To test how FXN and/or FDX2 affect the [2Fe–2S]-cluster assembly rate on ISCU2, we used circular dichroism spectroscopy to monitor changes in ellipticity at 430 nm as this wavelength is diagnostic of [2Fe–2S]^2+^-ISCU2 (Fig. 3d, Extended Data Fig. 5a–c and Extended Data Table 3). Without FXN present, samples containing 3 equivalents of FDX2 and an electron reduction system (FDXR and NADPH) showed a low Fe–S synthesis rate, matching the previous literature^18^. Only 0.5 equivalents of FXN were required to stimulate the assembly rate 5-fold; 1.5 and 3 FXN equivalents resulted in robust 6-fold and 13-fold stimulation, respectively. The ISCU2 cluster assembly rate was decreased 3-fold when 20 equivalents of FDX2 were added (FXN to FDX2 ratio of 3:20). These results confirm our prediction that excess FDX2 is inhibitory to the FXN-based stimulation of Fe–S assembly activity and are consistent with in vitro biochemical results from Want et al.^35^. These results indicate that in the case of low non-zero levels of FXN, lowering FDX2 levels so they are not in excess of FXN may increase overall Fe–S cluster production (Fig. 3e).

To gain biochemical insight into how our genetic suppressor mutations bypass the need for FXN, we tested the FDX2(E131K) variant in our in vitro cysteine desulfurase and Fe–S assembly assays. The addition of 20 equivalents or 60 equivalents of oxidized FDX2(E131K) resulted in a 5-fold activation of the SDA_ec_U cysteine desulfurase activity (Fig. 3c, purple). We eliminated the possibility that FDX(E131K)-based stimulation was an artefact of trace amounts of *E. coli* IscS that copurified with recombinant FDX2 by determining that samples containing 3 equivalents of ISCU and 60 equivalents of FDX2(E131K) but lacking SDA_ec_ had no observable cysteine desulfurase activity (data not shown). Next, we measured the Fe–S assembly rates on ISCU2 without FXN using 1.5 equivalents and 3 equivalents of FDX2(E131K) (Fig. 3d). The resulting 6–10-fold stimulation in activity further supports the conclusion that FDX2(E131K) can enhance Fe–S assembly without FXN. Unlike the inhibitory effect observed with excess FDX2 (Fig. 3d), samples containing 3 equivalents of FXN were further stimulated by excess FDX2(E131K). These results show that the FDX2(E131K) variant does not inhibit FXN-stimulated Fe–S cluster synthesis and raise the intriguing possibility that, in addition to weakening the NFS1–FDX2 interaction, some of our mutants may gain ‘frataxin-like’ stimulatory activity reminiscent of effects seen with FDX2 C-terminal mutations^9,35^.

To test whether FDX2 would also inhibit CFe–S cluster synthesis in vivo, we overexpressed wild-type FDX2 in K562 and 293T human cell lines. We observed that overexpression of wild-type FDX2 caused a very specific fitness defect, namely a growth defect in both cell lines in normoxia (21% O_2_) that was rescued under continuous hypoxia (1% O_2_) (Fig. 3f). By contrast, other members of Fe–S cluster machinery, such as NFS1 and ISCU2, did not produce adverse effects when overexpressed^7^, although notably overexpression of frataxin in vivo caused toxicity in mice^36,37^. Immunoblotting of K562 and 293T lysates from cells overexpressing green fluorescent protein (GFP) or FDX2 revealed that cells overexpressing FDX2 showed biochemical phenotypes of Fe–S cluster deficiency: we observed a reduction in lipoate synthesis by the Fe–S cluster-containing LIAS protein (Fig. 3g and Supplementary Data 2), and loss of Fe–S cluster-containing respiratory chain complexes I, II and IV (Fig. 3g). Many of these defects were partially or fully restored by growth under low oxygen tension (Fig. 3f,g and Supplementary Data 2). These results validate our in vitro experiments and suggest that frataxin loss in mammalian systems may also cause Fe–S cluster defects through stoichiometric excess of FDX2.

**Fig. 4:**
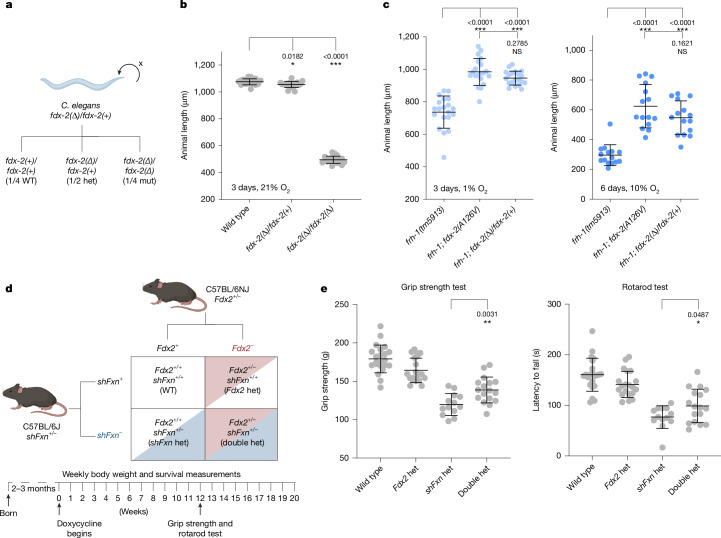
Partial loss of wild-type FDX2 activity suppresses frataxin mutants. **a**, *C. elegans* animals carrying a CRISPR–Cas9-generated *fdx-2* null mutation (mut) (8-base-pair deletion), predicted to produce no functional protein, were propagated as heterozygotes (het) and through self-fertilization produced wild types, heterozygotes and homozygous mutants. **b**, Growth of animals for 3 days at 21% oxygen. All animals were healthy and fertile except for *fdx-2* null homozygotes. The number of individual worms in each group was *n* = 20. **c**, Growth of animals for 3 days at 1% oxygen (left) or 6 days at 10% oxygen (right). Frataxin-null animals are rescued by a heterozygous null mutation in *fdx-2*. The number of individual worms in each group was *n* = 20 (left) or *n* = 12–15 (right). **d**, Schematic of mouse cross and experimental design. **e**, Grip strength (left) and latency to fall on a rotarod test (right) in mice on doxycycline treatment for 12 weeks. The number of individual mice in each group was: wild type *n* = 22, *Fdx2* het *n* = 20, *shFxn* het *n* = 13, double het *n* = 17. For all panels, statistical significance was calculated using one-way ANOVA followed by Dunnett’s (**b**) or Tukey’s (**c**) multiple comparison test, or a two-tailed unpaired *t*-test (**e**). Error bars represent mean ± s.d. *P < 0.05, **P < 0.01, ***P < 0.001. Schematics in **a** and **d** were created using BioRender (https://biorender.com).

**Extended Data Fig. 6:**
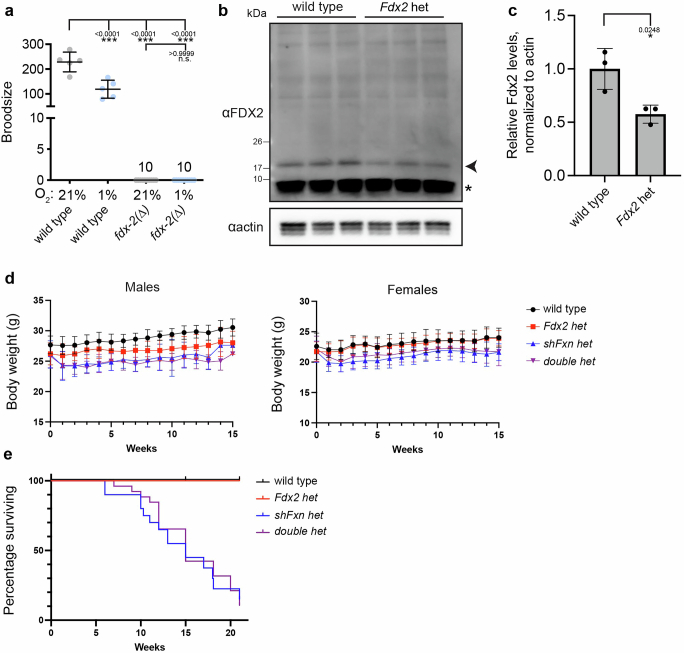
Partial loss of wild type FDX2 activity suppresses frataxin mutants. **a**, Broodsize of wild type and *fdx-2(null)* animals grown continuously at 21% or 1% oxygen. The number of individual worms in each group was: wild type 21% and 1% O_2_ n = 5, *fdx-2* 21% and 1% O_2_ n = 10. **b**, SDS-PAGE followed by western blot of brain tissue from wild type mice or mice heterozygous for *Fdx2* mutation. Arrow indicates FDX2, star indicates non-specific band. For gel source data, see Supplementary Data 1. **c**, Quantification of western blot using Fiji. n = 3 individual mice representing biological replicates for each group (**b,c**). **d**, Body weight measurements of male (left) and female (right) mice following Doxycycline treatment. The number of individual mice in each group was: wild type males n = 12, *Fdx2* het males n = 9, *shFxn* het males n = 10, double het males n = 12, wild type females n = 10, *Fdx2* het females n = 11, *shFxn* het females n = 10, double het females n = 14. Mean ± standard deviation is plotted, individual data points are available in Supplementary Table 4. **e**, Survival of mice following Doxycycline treatment. The number of individual mice in each group was: wild type n = 22, *Fdx2* het n = 20, *shFxn* het n = 20, double het n = 26. For all panels statistical significance was calculated using one-way ANOVA followed by Sidak’s (**a**) Multiple Comparison Test or a two-tailed unpaired t-test (**c**). Error bars represent mean ± standard deviation. n.s. = not significant, * = p value < 0.05, ** = p value < 0.01, *** = p value < 0.001.

These data predict that simply lowering the amount of FDX2 may boost Fe–S cluster production in a frataxin mutant, and so we attempted to rescue frataxin loss by removing one copy of wild-type *fdx-2* in *C. elegans*. We used CRISPR–Cas9 to generate a null allele of *fdx-2* that carried an 8-base pair deletion resulting in a frameshift and could be propagated as a heterozygote (Fig. 4a,b). These animals were homozygous lethal (Fig. 4b and Extended Data Fig. 6a), but the heterozygotes of *fdx-2(null)/fdx-2(+)* were viable and had a wild-type growth rate at all oxygen tensions (Fig. 4b). Of note, mutation in FDX2 can underlie autosomal recessive diseases, but parents are healthy, indicating humans can live with one copy of FDX2 (refs. ^38,39^). In support, the gnomAD database reveals that humans are tolerant of carrying one FDX2 loss-of-function allele^40^. Introduction of the heterozygous *fdx-2(null)/fdx-2(+)* into *frh-1(tm5913)* partially rescued the growth and developmental defects of frataxin mutants at all oxygen tensions and was not significantly different from the homozygous *fdx-2(A126V)* suppressor mutations isolated in our screen (Fig. 4c). These results indicate that the *fdx-2* suppressor mutations were dominant due to haploinsufficiency—simply losing one copy of *fdx-2* results in a mutant phenotype—and strongly support our model that in a frataxin mutant lowering the amount of NFS1 bound by FDX2 can restore Fe–S cluster biosynthesis.

To test whether lowering FDX2 levels could also improve disease phenotypes in a mouse model of Friedreich’s ataxia, we asked whether a 50% decrease in *Fdx2* gene dosage would suppress the movement disorders of a frataxin mouse mutant (Fig. 4d). We obtained mice from The Jackson Laboratory carrying a loss-of-function *Fdx2*^*em1Murr*^ allele generated as part of a large-scale mouse CRISPR mutagenesis effort (KOMP). As in *C. elegans* and humans, mouse *Fdx2* homozygotes are embryonic lethal, but heterozygotes are viable and appear healthy. We confirmed through western blotting that *Fdx2*^*em1Murr*^ heterozygous mice have roughly 50% wild-type levels of FDX2 protein in the brain (Extended Data Fig. 6b,c), validating the use of this mutant.

*Fdx2* heterozygotes were crossed with the *shFxn* mouse model of Friedreich’s ataxia^41^. This strain is heterozygous for doxycycline-inducible short-hairpin RNA (shRNA) against frataxin (*shFxn*) and following doxycycline treatment develops neurological defects and cardiomyopathy^41^. We have previously shown that chronic, continuous hypoxia can prevent and even reverse the ataxia phenotype in this shRNA-induced *Fxn* model, however, hypoxia cannot rescue the shortened lifespan due to cardiomyopathy^7,42^. From this single genetic cross we generated four genotypes of F_1_ hybrid mice: wild type, *Fdx2/+*, *shFxn* and *shFxn; Fdx2/+* (Fig. 4d). We began doxycycline treatment at 2–3 months of age to knockdown *Fxn* and assessed body weight and survival at weekly intervals and motor defects using grip strength and rotarod tests following 12 weeks of doxycycline treatment (Fig. 4d). We observed statistically significant improvement of ataxia phenotypes in the *shFxn; Fdx2/+* double mutants (Fig. 4e). Depletion of *Fdx2* did not affect body weight or lifespan in the *shFxn* background (Extended Data Fig. 6d,e), reminiscent of the effects of hypoxia in this model^7^. We conclude that lowering *Fdx2* levels partially ameliorates the neurological defects associated with frataxin loss in mice.

## Discussion

Through a large forward genetic screen in *C. elegans*, we identified frataxin suppressor mutations that map to the NFS1–FDX2 interface: *nfs-1(R244K)*,* fdx-2(E117K)*,* fdx-2(A126V)* and *fdx-2(P127S)* (see Extended Data Table 1 for homologous residues in humans). Screens in yeast have identified a frataxin suppressor mutation in the scaffold protein ISCU^15^ that bypasses the need for frataxin by accelerating cluster formation on the glutaredoxin GRX5 (ref. ^43^). However, such ‘bypass’ mutations have never been discovered in animals. On the basis of the published cryo-EM structure^9^, *nfs-1(R244K)* and *fdx-2(E117K)* probably disrupt salt-bridge interactions that ordinarily stabilize the NFS-1–FDX-2 interaction. Furthermore, our in vitro biochemical work suggests that at high concentrations FDX-2(E131K) may activate NFS-1 in a manner similar to frataxin^44,45^, reminiscent of FDX2 C-terminal mutations^9,35^ and possibly explaining why this specific *fdx-2* mutant was isolated from our screen. *fdx-2(A126V)* and *fdx-2(P127S)* mutations may also disrupt FDX-2 binding to NFS-1 through conformational changes of helix F, confer frataxin-like activity to FDX-2 or destabilize FDX-2 leading to less overall protein, which we observed for the A126V mutation in our TMT proteomics (Extended Data Fig. 3a). In principle any mutation that lowers FDX-2 levels could be a suppressor in this system, as heterozygous *fdx-2(null)* also partially rescued frataxin loss.

Our genetics and biochemistry studies collectively support a model in which frataxin and FDX2 compete for the same binding site on the Fe–S cluster complex in requisite, sequential steps of Fe–S cluster synthesis. FDX2 as an electron donor is always required for ISC synthesis, whereas FXN is conditionally required in normoxia. An imbalance in their relative levels—in which one is in excess relative to the other—can inhibit overall cluster formation. Indeed, most patients with Friedreich’s ataxia have 2–30% of residual frataxin protein^46^, and we show that the resulting excess FDX2 could lead to a decrease in frataxin-stimulated Fe–S cluster production. It is notable that gene therapy approaches for Friedreich’s ataxia involving frataxin overexpression have shown toxicity in mouse studies^36,37^ and paradoxically cause Fe–S cluster deficiency dependent on frataxin binding to NFS1 (ref. ^37^). The ability of frataxin to competitively inhibit FDX2 binding to NFS1 (ref. ^8^) explains why frataxin overexpression is toxic and motivates genetic therapies, such as partial knockdown of the essential gene FDX2, that restore the stoichiometric balance of frataxin and FDX2. Although our findings indicate the importance of relative protein levels, we do not know the precise in vivo stoichiometry, which is probably context-dependent and influenced by factors such as redox environment. In this study we also demonstrate that even in contexts of no frataxin protein (for example, *C. elegans* null mutant) or extremely low levels of frataxin (for example, doxycycline-inducible *shFxn* mouse), lowering FDX2 levels to even 50% of wild type is beneficial. This may be due to FDX2 actively inhibiting the sulfur transfer reaction from NFS1 to ISCU2 by affecting the NFS1 mobile S-transfer loop’s ability to interact with ISCU2. In this scenario lowering FDX2 levels may boost sulfur transfer chemistry and Fe–S cluster assembly due to an increase in NFS1 unbound by FDX2 (Fig. 3e). Our work is richly supported by the accompanying study by Want et al.^35^, who show that in the complete absence of frataxin, FDX2 is inhibitory to Fe–S synthesis in vitro, which can be overcome by point mutations that weaken the FDX2–NFS1 interaction.

Our work further clarifies how low oxygen conditions rescue defects in Fe–S cluster biosynthesis. Although hypoxia is known to rescue frataxin deficiency^7,24^, we show it also rescues growth and Fe–S cluster defects caused by FDX2 overexpression (Fig. 3f,g). One explanation is that hypoxia modulates the physical or functional interaction between FDX2 and the ISC assembly complex, consistent with evidence that FDX2 binding is sensitive to redox state^8,47^. Alternatively, hypoxia may complement the need for frataxin by stabilizing ISCs or oxygen-sensitive intermediates (for example, Fe^2+^, persulfides)^48^. In this model, frataxin provides ‘physical shielding’ of Fe–S cluster intermediates, whereas mitochondrial respiratory activity offers ‘respiratory shielding’ by consuming oxygen^49^. In the context of mutations causing reduced but non-zero Fe–S cluster levels or exposing intermediates to oxygen, hypoxia probably enhances Fe–S cluster synthesis and prolongs cluster half-life, enabling survival.

## Methods

### *C. elegans* strain maintenance and generation

*C. elegans* were propagated on nematode growth medium plates seeded with *Escherichia coli* strain OP50 (ref. ^50^). Some strains were provided by The Caenorhabditis Genetics Center, which is supported by the National Institutes of Health, Office of Research Infrastructure Programs (P40 OD010440). Frataxin *frh-1(tm5913)* animals were provided by the Mitani Laboratory through the National Bio-Resource Project of the MEXT, Japan. For a complete list of *C. elegans* strains used in this study see Supplementary Table 2. To generate mutants with CRISPR–Cas9, 30 pmol of *S. pyogenes* Cas9 (IDT) was injected into *C. elegans* gonads along with 90 pmol of transactivating CRISPR RNA (IDT), 95 pmol of CRISPR RNA (IDT), ssODN repair template (when applicable) and 40 ng µl^−1^ PRF4::*rol-6(su1006)* plasmid was used as a marker of successful injections^51^.

To screen for genetic suppressor mutations of *frh-1(tm5913)*, thousands of L4 animals were exposed to 47 mM ethyl methanesulfonate (Sigma M0880) for 4 h while rocking. Animals were then washed twice with M9 buffer and allowed to recover on standard nematode growth medium plates at 1% oxygen. F_2_ animals were produced by self-fertilization and are therefore homozygous for about 100 new mutations per F_2_ strain tested. Animals were bleach prepared as described below to generate a synchronized L1 stage population of mutagenized F_3_ animals, which were then dropped onto standard nematode growth medium plates at 10% oxygen. Plates were checked daily and F_3_ individuals capable of growing to adulthood were transferred onto new plates at 1% oxygen. Fertile isolates were retested using F_4_ or F_5_ progeny to confirm their phenotype and then genomic DNA for whole-genome sequencing was isolated using Gentra Puregene Tissue Kit (Qiagen, 158667). To identify candidate suppressor mutations screen isolates were whole-genome sequenced^52^. Lists of protein-altering mutations from each suppressor strain were then compared to identify genes with multiple mutant alleles. These candidate genes were then verified using targeted CRISPR–Cas9-based editing.

### Human cell lines maintenance and generation

K562 (female) and human embryonic kidney 293T (female) cells were obtained from American Type Culture Collection (ATCC) and cultured in DMEM (Gibco) with 25 mM glucose, 10% FBS (non-dialysed, Invitrogen), 1 mM pyruvate, 50 μg ml^−1^ uridine and 4 mM glutamine along with 100 U ml^−1^ penicillin/streptomycin under 5% CO_2_ at 37 °C. Cell lines were checked by ATCC profiling before purchase, and were tested to ensure absence of mycoplasma every 3 months. Cells were passaged every 2–3 days. The 293T cells were washed with PBS (Invitrogen) and dissociated using TrypLE (Gibco). For experiments with hypoxia, cells were placed in a Coy O_2_ Control Dual Hypoxia Chamber maintained at 37 °C, 1% O_2_ and 5% CO_2_ with appropriate humidity control.

For overexpression assays, complementary DNAs were either purchased from ORIGENE or custom synthesized from IDT. Constructs were cloned into pLYS6 bearing a Geneticin selection cassette, using the NheI and EcoRI sites. All plasmids were verified by sequencing. pMD2.G (Addgene 12259) and psPAX2 (Addgene 12260) were used for lentiviral packaging. For lentivirus production 2.5 × 10^6^ 293T cells were seeded in 5 ml in a T25-cm^2^ flask (1 lentivirus per flask). The following day the cells were transfected with 1 ml of transfection mixture, which contained 25 µl of Lipofectamine 2000 (Thermo Fisher Scientific), 3.75 µg of psPAX2, 2.5 µg of pMD2.G, 5 µg of lentiviral vector of interest and Opti-MEM medium (Gibco) up to 1 ml. The mixture was incubated at room temperature for 20 min before adding it to cells and then incubating for 6 h. Following the incubation, the media was replaced with fresh DMEM. Two days after transfection, media was collected, filtered through a 0.45-µm filter and stored at −80 °C. For infection, cells were seeded at 5 × 10^6^ cells per ml (K562) or 1 × 10^5^ (293T) in 2 ml per well in a six-well plate the day of infection. Cells were infected with virus and polybrene was added at a concentration of 1:1,000 final volume (Invitrogen). Cells were incubated for 48 h before being selected with Geneticin (500 µg ml^−1^) (Gibco) for 48 h.

### Mouse strains maintenance and generation

C57BL/6J-*shFxn* mice were provided by the Geshwind Laboratory at the University of California, Los Angeles^41^. C57BL/6NJ-*Fdx2*^*em1Murr/Murr*^ mice were bought from The Jackson Laboratory (strain no. 030192). C57BL/6J-*shFxn* mice and C57BL/6NJ-*Fdx2* mice were bred, pups were weaned and genotyped at roughly 25 days after birth. Mouse genotypes from tail biopsies were determined using quantitative PCR with specific probes designed for each gene (Transnetyx). All cages were provided with food and water ad libitum. Food and water were monitored daily and replenished as needed, and cages were changed weekly. A standard light–dark cycle of roughly 12 h light exposure was used at a temperature between 20 °C and 25 °C and humidity between 40% and 60%. Body weights were recorded weekly, and mice were humanely euthanized when they had lost 20% of peak body weight, in accordance with the American Veterinary Medical Association guidelines. For all experiments, animals were randomized on a 1/1 basis, balanced by age and sex. All animal studies were approved by the Subcommittee on Research Animal Care and the Institutional Animal Care and Use Committee of Massachusetts General Hospital.

For doxycycline knockdown, the average age of the animals at the start of experiments was 2–3 months. Doxycycline treatment followed the established optimal dosing protocol; 2 mg ml^−1^ doxycycline (Sigma) was added to the drinking water of all animals, which was changed weekly. In addition, animals were injected intraperitoneally with doxycycline twice a week, starting with 5 mg kg^−1^ body weight for 10 weeks followed by 10 mg doxycycline per kilogram of body weight at later timepoints.

### *C. elegans* and human cell culture assays

To measure *C. elegans* growth and development crowded plates of gravid animals were washed into tubes in M9 buffer (3 g KH_2_PO_4_, 6 g Na_2_HPO_4_, 5 g NaCl, 1 ml 1 M MgSO_4_, H_2_O to 1 l) and incubated with 20% bleach and 10% 5 M KOH for 5 min while vortexing. The resulting embryos were washed three times in M9 buffer and allowed to hatch overnight while rocking in M9. The following day arrested L1 animals were dropped onto *E. coli* OP50 plates and incubated at 20 °C. For assays in hypoxia (1% oxygen), animals were incubated in a hypoxic in vitro cabinet (Coy Laboratory Products, Inc.) at room temperature. To measure animal length, images were acquired using a ZEISS Axio Zoom V16 microscope with ZEN PRO software and the midline of individual animals was quantified in FIJI software. To measure *hsp-6::gfp* or *gst-4::gfp* fluorescence, L4 animals were mounted on agar pads, immobilized in levamisole, and imaged at ×70 magnification using a ZEISS Axio Zoom V16 microscope with ZEN PRO software. Fluorescent images were quantified by calculating the mean fluorescence along the midline of the intestine using FIJI software.

Human cell proliferation assays were performed between 8 days and 10 days following lentiviral infection. Cells were seeded at an initial density between 1 × 10^5^ and 2 × 10^5^ cells per ml (K562) or 1 × 10^5^ cells per well in a six-well plate (293T) and cultured for 3 days in either 21% or 1% oxygen tensions. Viable cell numbers were then determined using a Vi-Cell Counter (Beckman).

### Mouse movement and ataxia assays

#### Accelerating rotarod test

The amount of time for which mice could maintain their position on an accelerating rotating rod (Ugo Basile) was recorded. Mice were acclimated to the experimental room for at least 30 min before the start of the measurements. Rotarod parameters were as follows: acceleration of 5 rpm per minute and a maximum speed of 40 rpm. If mice used their body to grasp the rod (rather than walking on it) for more than 10 s, this time was recorded as time of fall. Each mouse was tested three times, and the trials were averaged.

#### Grip strength test

Fore and hind limbs grip strength was measured using a commercial dynamometer (Bioseb) over three consecutive efforts with 30 s of rest in between. The maximal effort (*g*) was used as absolute force (*g*) to assess whole-body strength. Mice were excluded if they refused the test (hanging more than 10 s on three repeated attempts). Each mouse was tested three times, and the trials were averaged.

### Western blotting

For *C. elegans* western blotting, SDS–PAGE western blots were performed with whole worm lysate, prepared by snap-freezing worm pellets of equal volume in liquid nitrogen and then mixing with NuPAGE LDS sample buffer and boiling at 100 °C for 20 min, followed by centrifugation at maximum speed for 10 min. Samples were run in 4–12% NuPAGE Bis-Tris gels for 2 h at 100 V. Gels were transferred to nitrocellulose membranes using the iBlot Dry Blotting system, and then blocked in 5% milk in Tris-buffered saline with Tween (TBST) for 1 h. Membranes were incubated in primary antibodies (anti-NDUFS3 Abcam ab14711; anti-ATP5A Abcam ab14748; anti-actin Abcam ab179467; Anti-Lipoic acid Sigma 437695) at 1:1,000 dilution overnight at 4 °C in 5% milk and TBST. For a complete list of antibodies used in this study see Supplementary Table 3. The following day membranes were washed for 1 h in TBST, incubated with secondary antibodies at 1:10,000 dilution for 1 h at room temperature in 5% milk and TBST, and then washed in TBST for another hour. Blots were developed using Pierce ECL Western Blotting Substrate (Fisher, 32209) and imaged with a GE Amersham Imager. All western blot experiments were repeated with at least *n *= 3 biological replicates.

For FDX2 western blot on mouse brain tissue, mice were euthanized under deep anaesthesia induced with 5% isoflurane and vital organs were collected and flash frozen in liquid nitrogen. Brains were pulverized in liquid nitrogen and total proteins were extracted using standard RIPA buffer plus protease inhibitor cocktail. Protein concentrations in tissue lysates were quantified using the BCA assay (Pierce), and western blot proceeded as detailed above.

For human cell culture protein immunoblotting, 2 × 10^6^–5 × 10^6^ K562 or 293T cells were gathered, washed in cold PBS and lysed for 15–30 min on ice in RIPA lysis buffer (Thermo Fisher) containing 1× HALT protease and phosphatase and Pierce Universal Nuclease for Cell Lysis (Thermo Fisher). Lysates were further clarified by centrifugation for 10 min at 10,000*g* at 4 °C. Supernatant was collected into fresh tubes, and protein concentration measured with Pier 660-nm protein assay (Thermo Fisher). Next, 30 µg was loaded per well in Novex Tris-Glycine 4–20% gels (Life Technologies). Gels were run for 55 min at 180 V and subsequently transferred onto a nitrocellulose membrane, 0.45 µM (BioRad). Membranes were stained with Ponceau S to check for adequate loading. Membranes were then blocked for 1–2 h with Odyssey Blocking Buffer (LI-COR Biosciences) at room temperature. Afterwards, membranes were incubated overnight at 4 °C with a solution of primary antibody diluted in Odyssey Blocking Buffer + 0.1% Tween-20 (anti-NFS1 1:1,000; anti-FDX2 1:1,000 (Atlas) or 1:100 (custom); anti-tubulin 1:5,000; anti-lipoic acid 1:1,000; anti-OXPHOS cocktail 1:250; anti-actin 1:5,000). For a complete list of antibodies used in this study see Supplementary Table 3. The next day, membranes were washed at room temperature three times for 3 min in TBST. The membrane was incubated with goat anti-rabbit or anti-mouse conjugated to IRDye800 or IRDye680 (LI-COR Biosciences) in a 1:1 solution of Odyssey Blocking Buffer (LI-COR Biosciences) and TBST. Membranes were incubated for 1 h at room temperature, and then washed three times in TBST for 10 min each. Membranes were then scanned for infrared signal using the Odyssey Imaging System (LI-COR Biosciences). Band Intensities were analysed with Image Studio LITE (LI-COR Biosciences).

### Mass spectrometry

Quantitative TMT proteomics was performed by the Thermo Fisher Center for Multiplexed Proteomics in the Department of Cell Biology at Harvard Medical School. From frozen worm pellets samples were lysed with trifluoroacetic acid and neutralized with 2 M Tris base. Lysates were reduced with tris(2-carboxyethyl)phosphine, alkylated with iodoacetimide and quenched with dithiothreitol. Initial protein amount was approximated based on turbidity measurement at 360 nm. Digestion was performed sequentially using Lys-C (1:50) and Trypsin (1:100) based on protease to protein ratio. Peptides were detected (MS1) in the Orbitrap, sequenced (MS2) in the ion trap and quantified (MS3) in the Orbitrap. Roughly 2 µl of each TMT-labelled sample was mixed to verify labelling success. Peptides were separated using a gradient of 3 to 27% 90% Acetonitrile in 0.1% formic acid over 180 min. MS2 spectra were searched using the Comet algorithm against a custom *C. elegans* + *E. coli* database containing its reversed complement and known contaminants. Peptide spectral matches were filtered to a 1% false discovery rate using the target–decoy strategy combined with linear discriminant analysis. Proteins were quantified only from peptides with a summed signal-to-noise threshold greater than 100, and only proteins with more than one peptide were used for analysis. Raw data available in Extended Data Table 1.

### In vitro biochemistry

#### Protein expression and purification

SDA_ec_, ISCU2 and FXN were expressed and purified as previously described^53^. FDX2 (amino acids 66–183, Q6P4F2) and FDX2 E131K were synthesized and inserted into a pET29b(+) vector containing a tobacco etch virus (TEV) protease cleavage His_6_-tag from Twist Bioscience after codon optimization for *E. coli* expression using the Vector Builder software. The pET29b(+) plasmids encoding native His-TEV-FDX2 and the His-TEV-FDX2 E131K variant were separately transformed into BL21(DE3) *E. coli* cells. Cells were grown in Terrific Broth (Research Products International) at 37 °C and induced with 0.4 mM isopropyl-β-d-thiogalactoside (IPTG). Following IPTG induction, the temperature was dropped to 16 °C, and the cells were gathered 16 h later. Cells were resuspended in lysis buffer (50 mM HEPES, 500 mM NaCl, 50 mM imidazole, 10 mM MgCl_2_, 10 mM CaCl_2_, pH 8.0), lysozyme (10 mg l^−1^, Sigma-Aldrich), protease inhibitor cocktail (10 mg l^−1^, Sigma-Aldrich) and 1 µg ml^−1^ DNase were added, and the cells were lysing by sonication (Branson Sonifier 450). The soluble fractions were loaded onto 2 × 5-ml HisTrap columns (Cytiva). FDX2 proteins were eluted using a linear gradient of buffer A (50 mM HEPES, 500 mM NaCl, 50 mM imidazole, pH 8.0) and buffer B (50 mM HEPES, 500 mM NaCl, 500 mM imidazole, pH 8.0) over 6 column volumes. The eluted proteins were digested with TEV protease (prepared in-house; 1:50, protease: protein) overnight at 4 °C, and the digested product was loaded onto a 5-ml HisTrap column (Cytiva) to remove the TEV protease and His-tag. The flow-through containing cleaved product was concentrated to 20 ml, diluted to 150 ml with Anion A buffer (50 mM HEPES, 10% glycerol (vol:vol), pH 7.5), and loaded onto an anion exchange column (26/20 POROS 50HQ, Applied Biosystems). FDX2 was eluted with Anion Buffer B (50 mM HEPES, 1 M NaCl, 10% glycerol (vol:vol), pH 7.5) using a linear gradient over 5 column volumes Brown fractions containing FDX2 were pooled and concentrated to 5 ml and loaded onto a HiLoad 16/100 Superdex 75 pg (Cytiva). The fractions corresponding to FDX2 were concentrated, flash frozen in liquid nitrogen and stored at −80 °C. The concentrations of FDX2 and FDX2 E131K were determined using an extinction coefficient of 11,000 M^−1^ cm^−1^ at 415 nm.

FDXR (amino acids 35–494, Q61578) was codon optimized and synthesized with a TEV-cleavage His_6_-tag using the same procedure as FDX2 and FDX2 E131K. To increase the solubility of FDXR, the pGro7 plasmid (Takara Bio) was transformed into BL21(DE3) cells along with the codon-optimized His-TEV-FDXR pET29b(+) plasmid, and cells were grown in autoinduction media (AI conditions^53^) at 37 °C. To induce expression of GroEL-GroES, 5 mg ml^−1^ arabinose was added to the media at inoculation. Cells were grown to an optical density at 600 nm of 1–2, 3.75 µM riboflavin was added to the media, and the temperature was dropped to 25 °C. The cells were grown for an extra 20 h and then collected and lysed using the same procedure as FDX2 and FDX2 E131K, except the buffering conditions also contained 10% glycerol for protein stability. The soluble fraction was supplemented with an extra 3.75 µM of riboflavin and loaded onto 2 × 5-ml HisTrap columns (Cytiva). The protein was eluted using a linear gradient of buffer C (50 mM HEPES, 500 mM NaCl, 10 mM imidazole pH 8.0, 10% glycerol) and buffer D (50 mM HEPES, 500 mM NaCl, 500 mM imidazole pH 8.0, 10% glycerol) over 6 column volumes. The eluted protein was digested with TEV protease (prepared in-house; 1:50, protease:protein) overnight at 4 °C in 50 mM HEPES, 500 mM NaCl, 10% glycerol, pH 8.0, and the digested product was loaded onto a 5-ml HisTrap column (Cytiva) to remove the TEV protease and His-tag. The flow-through containing cleaved product was concentrated to 5 ml and loaded onto a HiLoad 16/100 Superdex 75 pg (Cytiva). The fractions corresponding to FDXR were concentrated, frozen in liquid nitrogen and stored at −80 °C. The concentrations of FDXR were determined using an extinction coefficient of 11,300 M^−1^ cm^−1^ at 450 nm.

#### Cysteine desulfurase activity measurements

Velocities of each complex were determined using a modified methylene blue assay^43^. Protein complexes were prepared using 0.5 µM SDA_ec_, 1.5 µM ISCU2 and, when included, variable concentrations of FXN and FDX2 (0–30 µM). Each reaction mixture was incubated with 10 mM d,l-dithiothreitol and 5 µM Fe^2+^ for 15 min at 37 °C. Reactions were initiated with 2 mM L-cysteine and quenched with 20 mM* N*,*N*-diphenyl-*p*-phenylenediamine and 30 mM Fe^3+^ after 3 min. After quenching, samples were incubated for 20 min at 37 °C and centrifuged at 13,000 rpm for 5 min to synthesize methylene blue and remove precipitated protein. The absorbance of the supernatant was measured at 670 nm and converted to the concentration of sulfide produced using a standard curve. Activity measurements were conducted in an anaerobic glovebox (mBraun, 22 °C, <1 ppm O_2_; monitored by Teledyne Model 311 Oxygen Gas analyser).

#### Fe–S cluster assembly on ISCU2

All Fe–S assembly assays on ISCU2 were performed at room temperature using degassed buffer (50 mM Tris, 100 mM NaCl, pH 8.0) with a final reaction volume of 200 μl. Reactions were mixed in a 1-cm pathlength cuvette including the following components: 1 μM SDA_ec_, 100 μM ISCU2, 0–60 μM FXN, 0–60 μM FDX2 (or FDX2(E131K)), 1 μM FDXR, 500 μM Fe^2+^ and 500 μM NADPH. The cuvette was sealed with a rubber septa in an anaerobic chamber (<1 ppm O_2_) and parafilmed. Reactions were initiated with 500 µM l-cysteine using an air-tight syringe, and circular dichroism scans (Applied Photophysics Chirascan, V100) were monitored from 300 nm to 700 nm every 3 min for 30 min. The average change in ellipticity at 430 nm was plotted with time and fit to a linear equation using GraphPad Prism (*R*^2^ values greater than 0.95).

### Reporting summary

Further information on research design is available in the Nature Portfolio Reporting Summary linked to this article.

## Online content

Any methods, additional references, Nature Portfolio reporting summaries, source data, extended data, supplementary information, acknowledgements, peer review information; details of author contributions and competing interests; and statements of data and code availability are available at 10.1038/s41586-025-09821-2.

## Supplementary information

Supplementary Information This file contains Supplementary Data 1 and 2 and Tables 2 and 3.

Reporting Summary

Supplementary Table 1 Quantitative TMT proteomics from wild type, frataxin mutants and frataxin mutants with *fdx-2* or *nfs-1* suppressor mutations at 1% oxygen or shifted from 1% to 21% oxygen for 2 days (raw data).

Supplementary Table 4 Raw individual data values corresponding to every data point in the main figures and Extended Data figures.

## Figures and Tables

**Extended Data Table 1:**
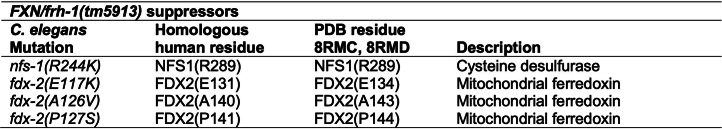
List of mutations reported in this study isolated from frataxin suppressor screens

Human *FDX2* gene model used for homology is NP_001384335.1.

**Extended Data Table 2:**
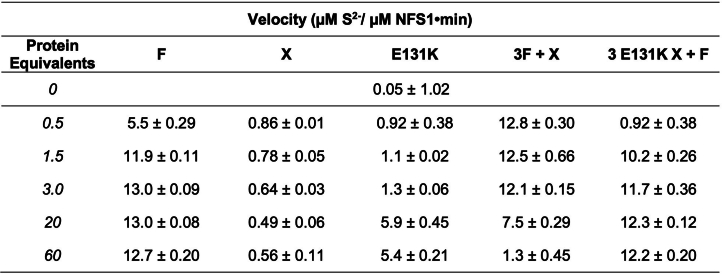
Cysteine desulfurase activity of SDA_ec_U complexes with variable ratios of FXN (F), FDX2 (X), and FDX2(E131K)

**Extended Data Table 3:**
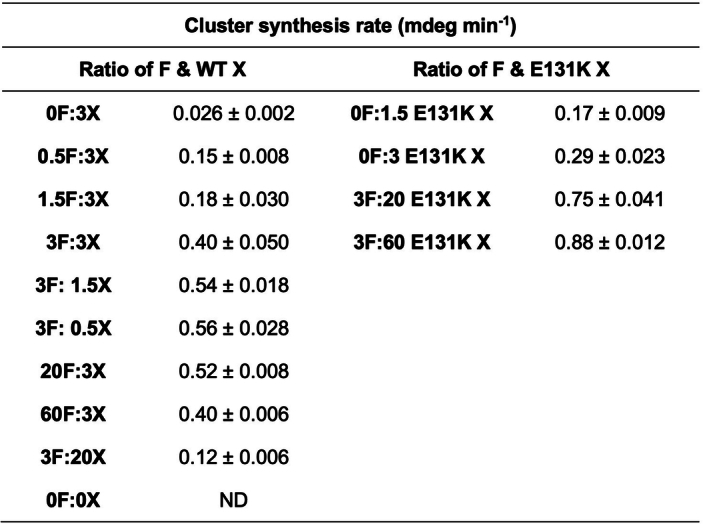
Kinetic data for [2Fe-2S]-cluster assembly on ISCU2 with variable ratios of FXN (F) and WT FDX2 (X) or FDX2(E131K)

ND, not detected. Ratios indicate protein equivalents compared to SDA_ec_.

## Data Availability

All datasets supporting the findings of this study are available within the paper and Supplementary Information.
