# Supplementary material for: Mutations in mitochondrial ferredoxin FDX2 suppress frataxin deficiency
Source: Nature. 2025 Dec 10;649(8097):713–20. doi: 10.1038/s41586-025-09821-2 (PMC12804076; doi:10.1038/s41586-025-09821-2)
Supplement: Supplementary file 1 — This file contains Supplementary Data 1 and 2 and Tables 2 and 3. [file 41586_2025_9821_MOESM1_ESM.pdf]

nature portfolio

https://doi.org/10.1038/s41586-025-09821-2

# Supplementary information

# Mutations in mitochondrial ferredoxin FDX2 suppress frataxin deficiency

In the format provided by the authors and unedited

**a**

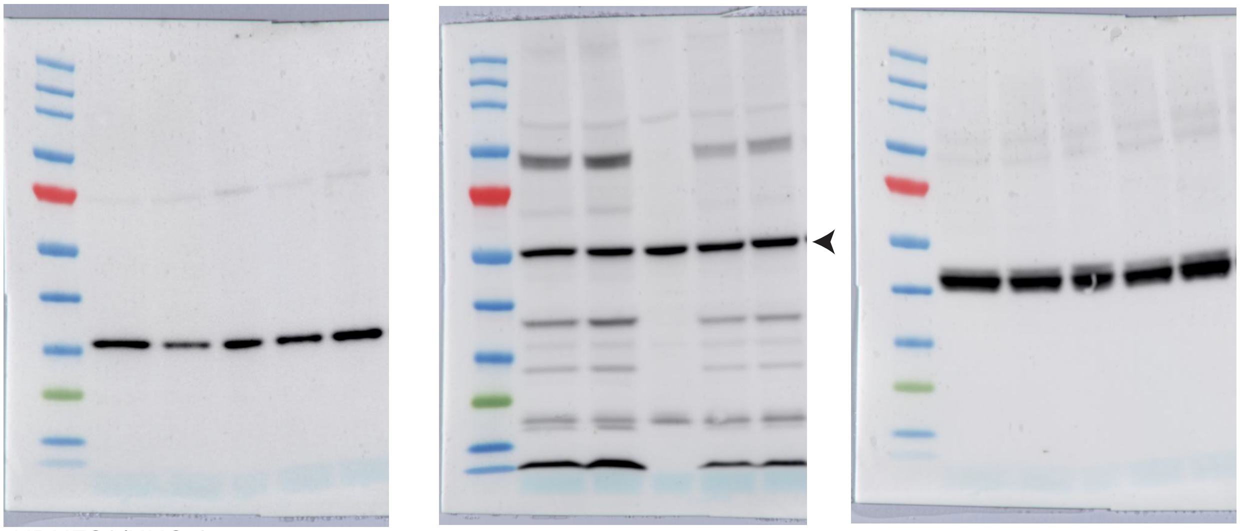

αNDUFS3/NUO-2 αLipoic acid αActin

**b**

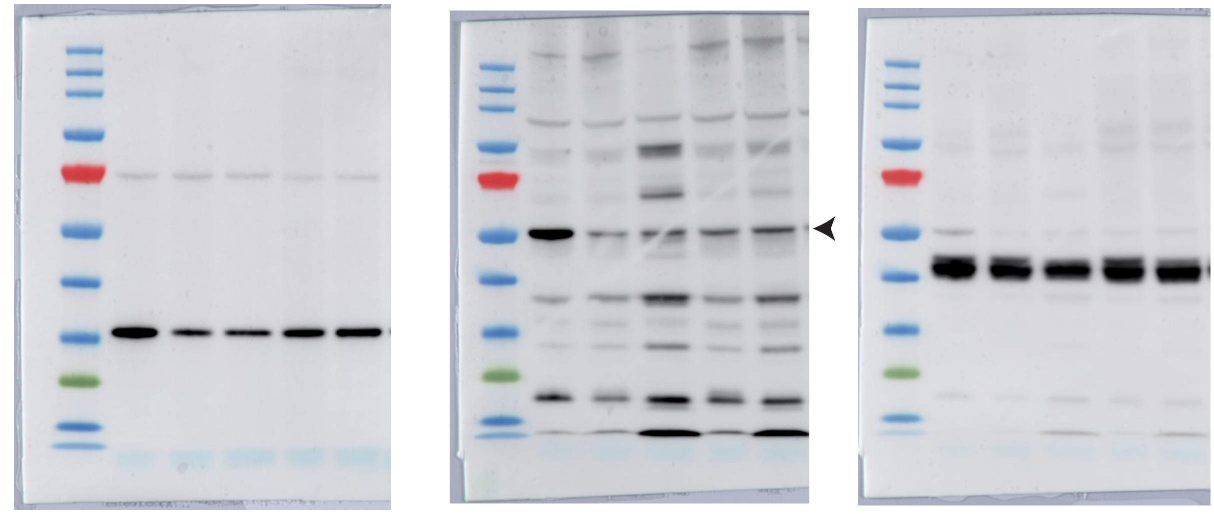

αNDUFS3/NUO-2 αLipoic acid αActin

**c**

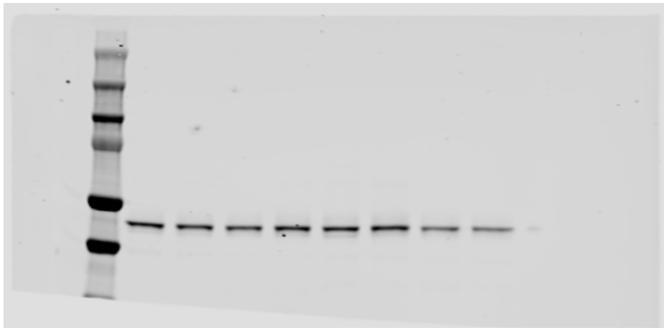

αNFS1

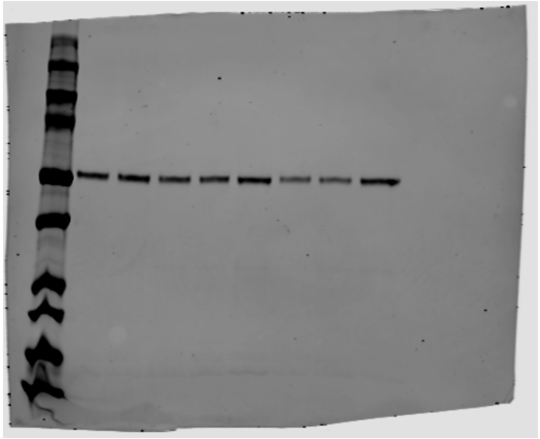

αTubulin

**d**

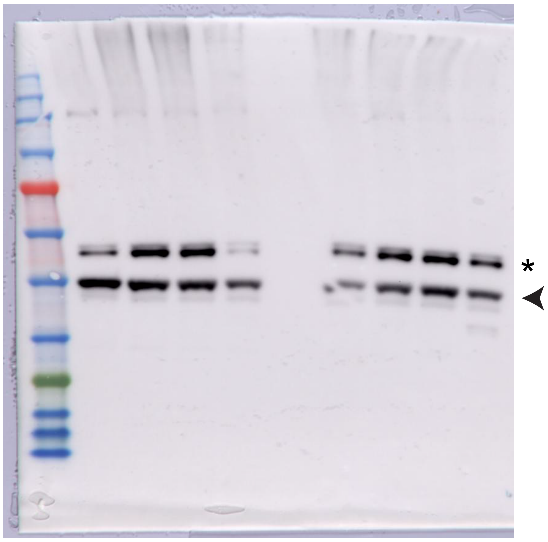

αActin

**e**

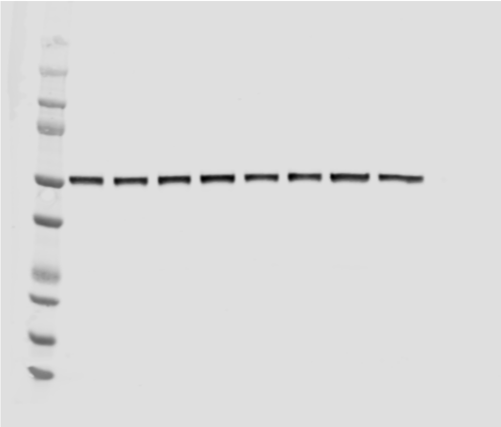

αtubulin

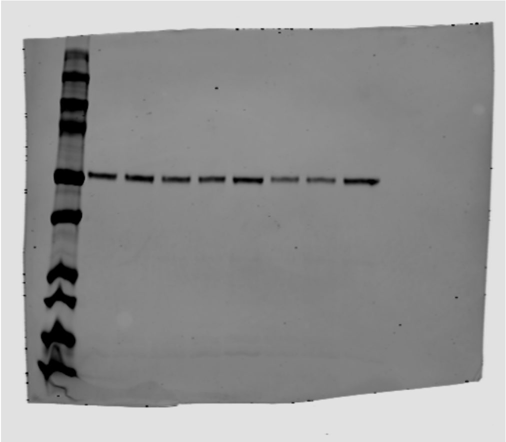

αtubulin

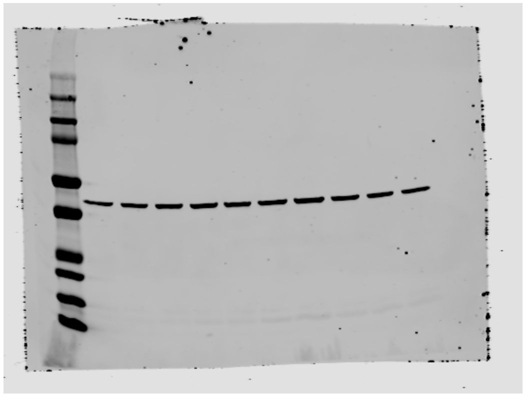

αActin

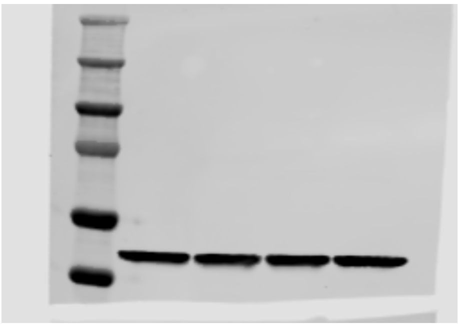

αActin

**Supplementary Data 1:** Source data of full western blot gels for Extended Data Figure 3b (**a**), Extended Data Figure 3c (**b**), Figure 3g (**c**), Extended Data Figure 4a (**d**), and Supplementary Data 2 (**e**).

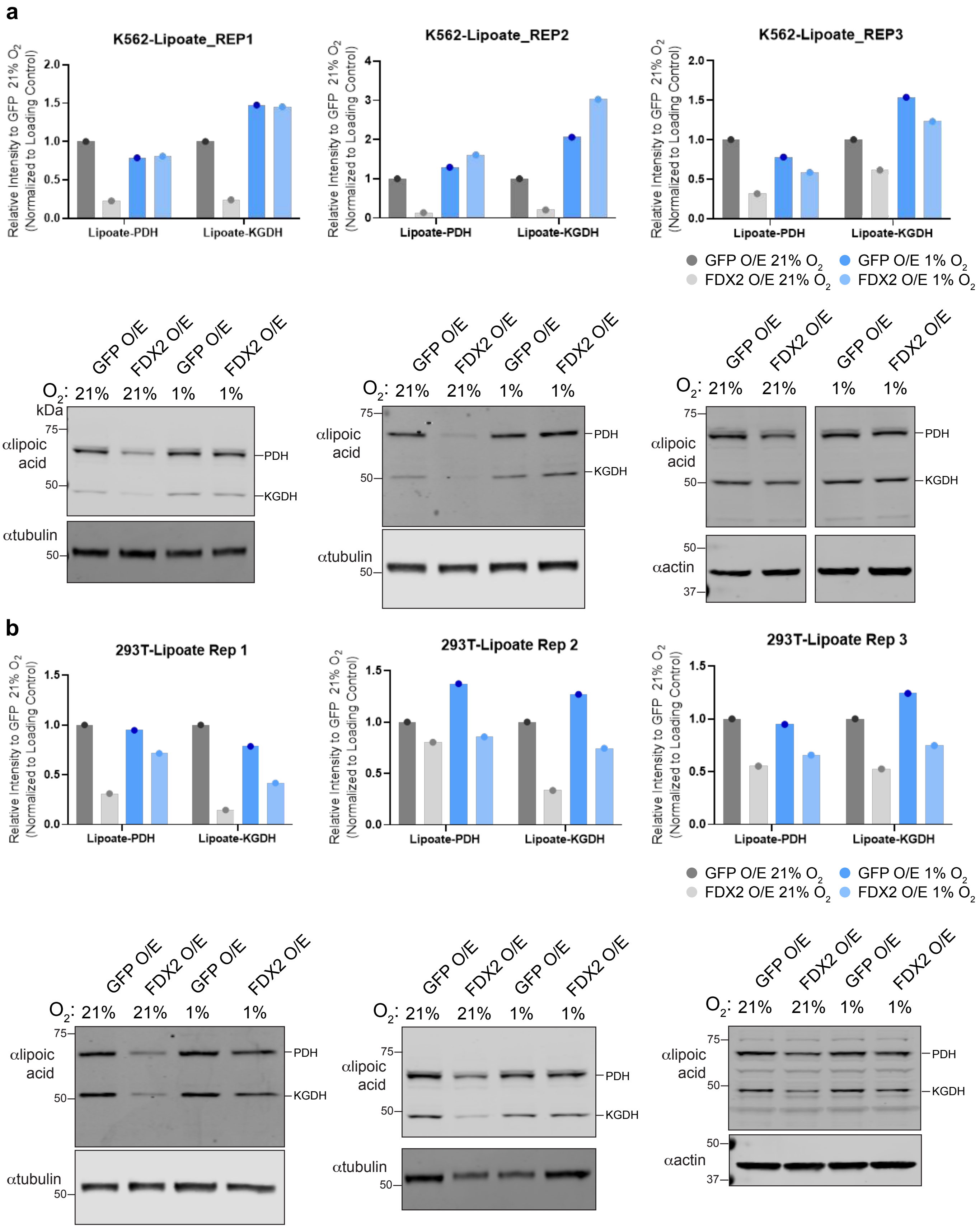


**Supplementary Data 2:** Excess FDX2 is detrimental to NFS1 activity and ISC biosynthesis. **a,b,** Individual biological replicates of immunoblots for lipoic acid and loading control on K562 (**a**) and 293T (**b**) cell lysates. n = three biological replicates, and each graph represents quantification of n = 1 individual blot. For gel source data, see Supplementary Data 1.

**Supplementary Methods Table 2:** List of *C. elegans* strains used in this study

| Strain | Genotype | Source |
|---|---|---|
| GR3080 | *frh-1(tm5913); zcIs13[hsp-6::GFP] V* | Ast et al. 2019 |
| GR3073 | *frh-1(tm5913) II* | Ast et al. 2019, derived from FX16013 |
| CL2166 | *dvIs19[gst-4::gfp] III* | CGC |
| N2 | wild type | CGC |
| SJ4100 | *zcIs13[hsp-6::GFP] V* | CGC |
| GR3616 | *nfs-1(mg800[R244K]) I* | This study |
| GR3617 | *fdx-2(mg801[E117K]) IV* | This study |
| GR3618 | *fdx-2(mg802[A126V]) IV* | This study |
| GR3619 | *fdx-2(mg803[P127S]) IV* | This study |
| GR3620 | *nfs-1(mg800[R244K]); frh-1(tm5913)* | This study |
| GR3621 | *frh-1(tm5913); fdx-2(mg801[E117K])* | This study |
| GR3622 | *frh-1(tm5913); fdx-2(mg802[A126V])* | This study |
| GR3623 | *frh-1(tm5913); fdx-2(mg803[P127S])* | This study |
| GR3624 | *frh-1(tm5913)/mIn1; mgEx865[Prpl-28::NDI + ofm-1::gfp]* | This study |
| GR3625 | *nfs-1(mg800[R244K]) I; frh-1(tm5913); zcIs13[hsp-6::GFP] V* | This study |
| GR3626 | *frh-1(tm5913); fdx-2(mg802[A126V]) IV; zcIs13[hsp-6::GFP] V* | This study |
| GR3627 | *frh-1(tm5913); dvIs19[gst-4::gfp] III* | This study |
| GR3628 | *nfs-1(mg800[R244K]) I; frh-1(tm5913); dvIs19[gst-4::gfp] III* | This study |
| GR3629 | *frh-1(tm5913); dvIs19[gst-4::gfp] III; fdx-2(mg802[A126V]) IV* | This study |
| GR3630 | *nfs-1(mg800[R244K]); fdx-2(mg802[A126V])* | This study |
| GR3631 | *fdx-2(mg804[8bp deletion])/nT1 IV; +/nT1 V* | This study |
| GR3632 | *frh-1(tm5913); fdx-2(mg804[8bp deletion])/nT1 IV; +/nT1 V* | This study |
| JDM1010 | *frh-1(tm5913); fdx-2(mg803[P127S]); zcIs13[hsp-6::GFP] V* | This study |

**Supplementary Methods Table 3:** List of antibodies used in this study

| Antigen | Cat. Number | Vendor |
|---|---|---|
| NDUFS3 | ab14711 | Abcam |
| ATP5A | ab14748 | Abcam |
| OXPHOS | ab110411 | Abcam |
| NFS1 | sc-365308 | Santa Cruz Biotechnology |
| FDX2 | HPA043986 | Atlas |
| FDX2 | Custom Synthesized | Thermo Fisher |
| Tubulin | MA5-16308 | Invitrogen |
| Actin | ab179467 | Abcam |
| Actin | ab8227 | Abcam |
| Actin | 8H10D10 | Cell Signaling |
| Lipoic Acid | 437695 | EMD Millipore |
| Lipoic Acid | ab58724 | Abcam |
