# Supplementary material for: Mutations in mitochondrial ferredoxin FDX2 suppress frataxin deficiency
Source: Nature. 2025 Dec 10;649(8097):713–20. doi: 10.1038/s41586-025-09821-2 (PMC12804076; doi:10.1038/s41586-025-09821-2)
Supplement: Supplementary file 2 — Reporting Summary [file 41586_2025_9821_MOESM2_ESM.pdf]

Corresponding author(s): Meisel, Ruvkun, Mootha

Last updated by author(s): Sep 22, 2025

Reporting Summary

Nature Portfolio wishes to improve the reproducibility of the work that we publish. This form provides structure for consistency and transparency in reporting. For further information on Nature Portfolio policies, see our Editorial Policies and the Editorial Policy Checklist.

Statistics

For all statistical analyses, confirm that the following items are present in the figure legend, table legend, main text, or Methods section.

n/a	Confirmed	
☐	☒	The exact sample size (*n*) for each experimental group/condition, given as a discrete number and unit of measurement
☐	☒	A statement on whether measurements were taken from distinct samples or whether the same sample was measured repeatedly
☐	☒	The statistical test(s) used AND whether they are one- or two-sided<br>*Only common tests should be described solely by name; describe more complex techniques in the Methods section.*
☐	☒	A description of all covariates tested
☐	☒	A description of any assumptions or corrections, such as tests of normality and adjustment for multiple comparisons
☐	☒	A full description of the statistical parameters including central tendency (e.g. means) or other basic estimates (e.g. regression coefficient) AND variation (e.g. standard deviation) or associated estimates of uncertainty (e.g. confidence intervals)
☐	☒	For null hypothesis testing, the test statistic (e.g. *F*, *t*, *r*) with confidence intervals, effect sizes, degrees of freedom and *P* value noted<br>*Give P values as exact values whenever suitable.*
☒	☐	For Bayesian analysis, information on the choice of priors and Markov chain Monte Carlo settings
☒	☐	For hierarchical and complex designs, identification of the appropriate level for tests and full reporting of outcomes
☒	☐	Estimates of effect sizes (e.g. Cohen's *d*, Pearson's *r*), indicating how they were calculated

Our web collection on statistics for biologists contains articles on many of the points above.

Software and code

Policy information about availability of computer code

Data collection	ZEN Pro, FIJI
Data analysis	GraphPad Prism

For manuscripts utilizing custom algorithms or software that are central to the research but not yet described in published literature, software must be made available to editors and reviewers. We strongly encourage code deposition in a community repository (e.g. GitHub). See the Nature Portfolio guidelines for submitting code & software for further information.

Data

Policy information about availability of data

All manuscripts must include a data availability statement. This statement should provide the following information, where applicable:

- Accession codes, unique identifiers, or web links for publicly available datasets
- A description of any restrictions on data availability
- For clinical datasets or third party data, please ensure that the statement adheres to our policy

The authors declare that all datasets supporting the findings of this study are available within the paper and its supplementary information files. Additional raw data supporting the finding of this paper are available from the corresponding authors upon reasonable request.

Research involving human participants, their data, or biological material

Policy information about studies with human participants or human data. See also policy information about sex, gender (identity/presentation), and sexual orientation and race, ethnicity and racism.

Reporting on sex and gender	N/A
Reporting on race, ethnicity, or other socially relevant groupings	N/A
Population characteristics	N/A
Recruitment	N/A
Ethics oversight	N/A

Note that full information on the approval of the study protocol must also be provided in the manuscript.

Field-specific reporting

Please select the one below that is the best fit for your research. If you are not sure, read the appropriate sections before making your selection.

☒ Life sciences ☐ Behavioural & social sciences ☐ Ecological, evolutionary & environmental sciences

For a reference copy of the document with all sections, see nature.com/documents/nr-reporting-summary-flat.pdf

Life sciences study design

All studies must disclose on these points even when the disclosure is negative.

Sample size	Sample sizes were not predetermined. Sample sizes for all experiments are stated and defined in figure legends. In general we sought to include between 12-20 individual animals for each experimental group and repeated each experiment in biological triplicate.
Data exclusions	No data was excluded in this study.
Replication	All experiments were completed in biological triplicate with the exception of TMT Proteomics.
Randomization	Experiments were not randomized, biological replicates were treated identically.
Blinding	Investigators were not blinded, biologiocal replicates were treated identically.

Reporting for specific materials, systems and methods

We require information from authors about some types of materials, experimental systems and methods used in many studies. Here, indicate whether each material, system or method listed is relevant to your study. If you are not sure if a list item applies to your research, read the appropriate section before selecting a response.

Materials & experimental systems

n/a	Involved in the study	
☐	☒	Antibodies
☐	☒	Eukaryotic cell lines
☒	☐	Palaeontology and archaeology
☐	☒	Animals and other organisms
☒	☐	Clinical data
☒	☐	Dual use research of concern
☒	☐	Plants

Methods

n/a	Involved in the study	
☒	☐	ChIP-seq
☒	☐	Flow cytometry
☒	☐	MRI-based neuroimaging

Antibodies

Antibodies used	NDUFS3 (Abcam, ab14711, 1:1000), ATP5A (Abcam, ab14748, 1:1000), OXPHOS (Abcam, ab110411, 1:250), NFS1 (Santa Cruz, sc-365308, 1:1000), FDX2 (Atlas, HPA043986, 1:1000), Tubulin (Invitrogen, MA5-16308, 1:5000), Actin (Abcam, ab179467, 1:10,000), Lipoic Acid (Sigma, 437695, 1:1000)

Validation

Manufacturer's Websites:
This Anti-Lipoic Acid Rabbit pAb is validated for use in ELISA, Frozen Sections, Immunoblotting, Paraffin Sections for the detection of Lipoic Acid.
Mouse Monoclonal NDUFS3 antibody. Suitable for Flow Cyt, WB and reacts with Human, Mouse, Cow, Rat samples.
Anti-ATP5A antibody [15H4C4] - Mitochondrial Marker (ab14748) is a mouse monoclonal antibody detecting ATP5A in Western Blot, Flow Cytometry, IHC-P, ICC/IF. Suitable for Cow, Drosophila melanogaster, Human, Mouse, Rat.
Total OXPHOS Human Western Blot Antibody Cocktail ab110411 is an optimized cocktail of antibodies for analyzing relative OXPHOS complex levels in Human mitochondria by Western Blot.
Anti-Actin antibody [EPR16769] (ab179467) is a rabbit monoclonal antibody detecting Actin in Western Blot, Flow Cytometry (Intra), Flow Cytometry, IP, IHC-P, ICC/IF. Suitable for Chicken, Human, Mouse, Rat.
beta Tubulin Loading Control Monoclonal Antibody (BT7R) Advanced Verification
Atlas Antibodies Sigma-Aldrich FDX2 HPA043986, Western Blot Validated Enhanced - Recombinant expression
NFS1 Antibody (B-7) is a mouse monoclonal IgG1 kappa light chain antibody that detects NFS1 protein of mouse, rat, and human origin by western blotting (WB), immunoprecipitation (IP), immunofluorescence (IF), immunohistochemistry, and enzyme-linked immunosorbent assay (ELISA).

Eukaryotic cell lines

Policy information about cell lines and Sex and Gender in Research

Cell line source(s)	K562 (female) and HEK293T (female) cells were obtained from ATCC
Authentication	Cells were authenticated by ATCC. No authentication was performed in this study.
Mycoplasma contamination	All cell lines tested negative for mycoplasma.
Commonly misidentified lines (See ICLAC register)	None used in this study.

Animals and other research organisms

Policy information about studies involving animals; ARRIVE guidelines recommended for reporting animal research, and Sex and Gender in Research

Laboratory animals	Many Caenorhabditis elegans animals were used in this study, see methods for a complete list of strains. Their ages range from larval stage 1 to adults. C57BL/6J-shFxn mice (provided by the Geshwind Laboratory at UCLA) and C57BL/6NJ-Fdx2em1Murr/Murr mice (bought from the Jackson laboratory strain No: 030192) were used in this study. Mice began Dox treatment at 2-3 months of age and were phenotyped over the next 12 weeks.
Wild animals	No wild animals were used in this study.
Reporting on sex	All C. elegans experiments were performed on hermaphrodite worms. For mouse studies both males and females were included in the experimental design.
Field-collected samples	No field-collected samples were used in this study.
Ethics oversight	Massachusetts General Hospital Institutional Animal Care and Use Committee.

Note that full information on the approval of the study protocol must also be provided in the manuscript.

Plants

Seed stocks	*Report on the source of all seed stocks or other plant material used. If applicable, state the seed stock centre and catalogue number. If plant specimens were collected from the field, describe the collection location, date and sampling procedures.*
Novel plant genotypes	*Describe the methods by which all novel plant genotypes were produced. This includes those generated by transgenic approaches, gene editing, chemical/radiation-based mutagenesis and hybridization. For transgenic lines, describe the transformation method, the number of independent lines analyzed and the generation upon which experiments were performed. For gene-edited lines, describe the editor used, the endogenous sequence targeted for editing, the targeting guide RNA sequence (if applicable) and how the editor was applied.*
Authentication	*Describe any authentication procedures for each seed stock used or novel genotype generated. Describe any experiments used to assess the effect of a mutation and, where applicable, how potential secondary effects (e.g. second site T-DNA insertions, mosiacism, off-target gene editing) were examined.*
